# A stochastic approach for parameter optimization of feature detection algorithms for non-target screening in mass spectrometry

**DOI:** 10.1007/s00216-024-05425-3

**Published:** 2024-07-12

**Authors:** Mohammad Sadia, Youssef Boudguiyer, Rick Helmus, Marianne Seijo, Antonia Praetorius, Saer Samanipour

**Affiliations:** 1 Institute for Biodiversity and Ecosystem Dynamics, University of Amsterdam, Amsterdam, The Netherlands; 2 Van‘T Hoff Institute for Molecular Sciences (HIMS), University of Amsterdam, Amsterdam, The Netherlands

**Keywords:** Non-target screening, Open-source algorithm, Feature detection, Parameter optimization

## Abstract

Feature detection plays a crucial role in non-target screening (NTS), requiring careful selection of algorithm parameters to minimize false positive (FP) features. In this study, a stochastic approach was employed to optimize the parameter settings of feature detection algorithms used in processing high-resolution mass spectrometry data. This approach was demonstrated using four open-source algorithms (OpenMS, SAFD, XCMS, and KPIC2) within the patRoon software platform for processing extracts from drinking water samples spiked with 46 per- and polyfluoroalkyl substances (PFAS). The designed method is based on a stochastic strategy involving random sampling from variable space and the use of Pearson correlation to assess the impact of each parameter on the number of detected suspect analytes. Using our approach, the optimized parameters led to improvement in the algorithm performance by increasing suspect hits in case of SAFD and XCMS, and reducing the total number of detected features (i.e., minimizing FP) for OpenMS. These improvements were further validated on three different drinking water samples as test dataset. The optimized parameters resulted in a lower false discovery rate (FDR%) compared to the default parameters, effectively increasing the detection of true positive features. This work also highlights the necessity of algorithm parameter optimization prior to starting the NTS to reduce the complexity of such datasets.

**Graphical Abstract:**

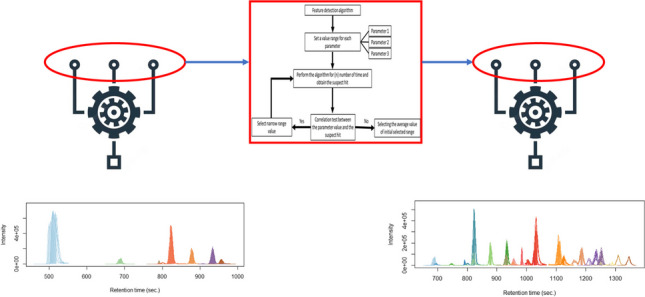

**Supplementary Information:**

The online version contains supplementary material available at 10.1007/s00216-024-05425-3.

## Introduction

An immense number of chemicals are produced globally and the inevitable release of synthetic chemicals to the environment increases the likelihood for negative impacts on human health and ecosystems [1, 2]. Conventional monitoring methods (target analysis) only cover a small part of the chemicals in use, which is a major impediment to evaluating chemical exposure and risks. Nowadays, suspect and non-target screening (NTS) employing high-resolution mass spectrometry (HRMS) are increasingly being used to assess the presence of a wider range of compounds in the environment [3]. The HRMS instruments, coupled with liquid and gas chromatography, allow for the untargeted detection of thousands of compounds that are compatible with the extraction, separation, and ionization methods involved [4–6].

In recent years, NTS of chemicals in the environment using HRMS has grown rapidly in the research community [7–12]. NTS is a bottom-up approach and considers all signals detected in full-scan HRMS without prior information [13]. However, applying NTS requires an automated process for screening all the experimental data since it is time-consuming to go through the data manually. Automation may give the illusion of reproducibility across users and data analysis workflows. However in reality, current approaches, each with different strengths and weaknesses, have been shown to deliver different results on identical datasets due to differences in underlying algorithms and parameter selection therein [14]. Harmonization is therefore required to enable comparison between different studies.

**Fig. 1:**
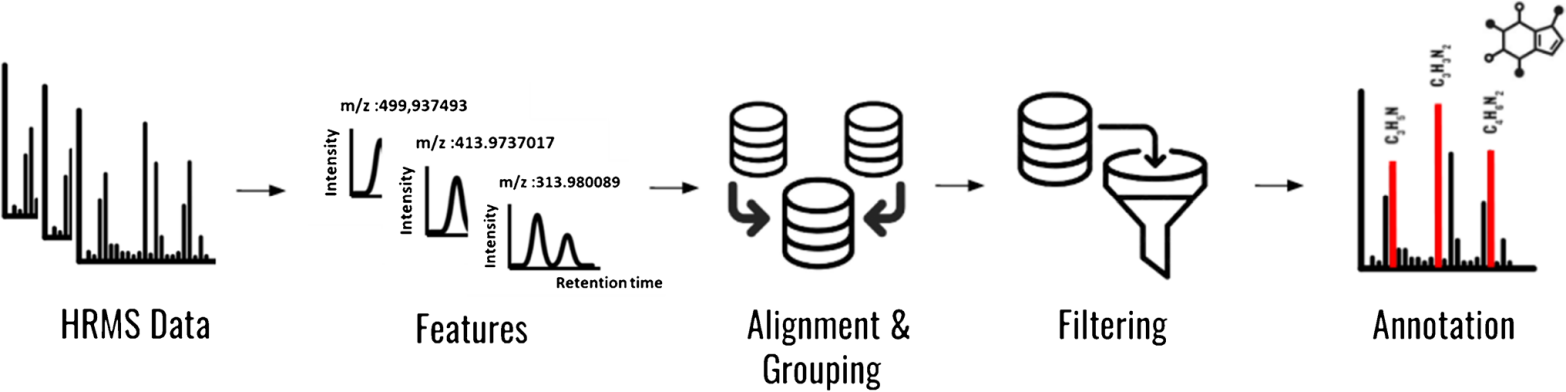
Scheme of a typical non-target screening (NTS) workflow. From left to right: (1) data acquisition and data pretreatment; (2) chromatographic peak detection; (3) feature alignment-and-grouping process, where features across samples are corrected for chromatography shifts and are combined across samples that are considered as equivalent respectively; (4) rule-based filtering and prioritization of feature groups; (5) formula generation and compound annotation of the filtered feature groups

A typical NTS data processing workflow consists of several steps (Fig. 1). Firstly, so-called features need to be extracted from the HRMS data. A feature is defined as a collection of data that holds a unique combination of a mass-to-charge ratio (m/z), peak area intensity, and retention time [15–17]. The next step is the alignment-and-grouping of the features in the samples and sample replicates where correction for chromatographical differences and the combining of features across replicate samples that are considered equivalent are performed respectively. Then the feature groups are filtered by subtraction of blank peaks and noise. Finally, the last step is annotation to elucidate chemical identities where formulas and chemical structures are generated based on the collected data [5, 16–18]. Suspect screening can be performed to reduce the complexity and difficulty of NTS using a suspect list that holds the exact masses of known chemicals to screen against collected features for similarity.

NTS can be performed using various closed- and open-source software tools. Closed-source commercial software tools use concealed algorithms and can implement the full NTS workflow for example DataAnalysis (Bruker), UNIFI (Waters), and Compound Discover (Thermo). Closed source software is normally applicable only to a vendor-specific data format, resulting in difficulties in data sharing and reproducibility. In contrast, many open-source software tools enable data sharing and implementation of reproducible workflows. Examples of such open-source software are OpenMS [19], XCMS [20], KPIC2 [21], SAFD [22], and MZmine [23], many of which are implemented in the open-source platform patRoon for environmental mass spectrometry–based NTS [16]. However, these algorithms, independent from their source, must be optimized for a given analysis to maximize the true positive rate while maintaining low rate of false positives [24, 25]. Employing differing algorithms or using the same algorithm with different settings may generate different results for the same data [26–30].

Feature detection is a crucial step in the NTS workflow and can increase uncertainty due to the generation of numerous false positive (FP) features, which arise from noise, artifacts, or mathematical effects rather than from actual chemicals [30]. The purpose of the feature detection step is to identify all signals caused by true features, while avoiding the detection of false features (e.g., noise and/or background signal), which is the common challenge for feature detection algorithms [12]. These algorithms often employ complex approaches using characteristic defined shape properties, such as smoothed second-derivatives, local maxima and minima, or wavelet models [23, 28, 31]. They typically considered both the time and mass domains, assuming a Gaussian-like distribution, and use both centroided and profile data.

However, the capabilities of existing algorithms are limited when it comes to identifying features with low intensity, non-Gaussian peak shapes, or those with poor baseline resolution [27, 32]. Additionally, most available algorithms require users to fine-tune a number of nonintuitive or “black box” input parameters, which limits their use to experts and can have unpredictable consequences for data quality [17, 32, 33]. Proper optimization of these algorithms is essential to achieve optimal performance in each step of peak detection and to reduce the detection rate of FP features, particularly when applied to complex environmental samples in NTS.

This study aims to design and evaluate an optimization method for feature detection algorithm parameters, which can be also applied to feature alignment-and-grouping algorithm. This work was performed with patRoon, an R-based open-source software platform for performing NTS workflows [16, 17]. Four different open-source algorithms: OpenMS [19], XCMS [20], KPIC2 [21], and SAFD [22], available within the patRoon platform, were used to evaluate the designed method and compare the optimized and default parameters for the outcome of each algorithm. A closed-source algorithm (DataAnalysis, Bruker Daltonics) was included as a comparison with the open-source algorithms. Our approach is applied to drinking water samples spiked a total of 46 per- and polyfluoroalkyl substances (PFAS) in order to optimize the feature finding and feature alignment-and-grouping algorithms. While our strategy focuses on maximizing the detection of the true-positive features (suspect hits) through optimizing the algorithm performance, it also considers the need to balance this with the minimization of false positives to avoid introducing extra complexities to the NTS workflow.

## Method and material

### Chemicals and standards

Native of 46 PFAS standards (Table S1) were purchased from Wellington Laboratories (Campro Scientific, The Netherlands), with the exception of N-deuteriomethylperfluoro-1-n-octanesulfonamidoacetic acid-d3 (N-MeFOSAA-d3, > 99%) and N-ethylperfluoro-1-n-octanesulfonamidoacetic acid-d5 (N-EtFOSAA-d5, > 99%) which were purchased from Chiron (Trondheim, Norway); trifluoroacetic acid (TFA, > 99%) and perfluoropropanoic acid (PFPrA, > 97%) were purchased from Sigma-Aldrich, The Netherlands; perfluoroethane sulfonic acid (PFEtS, > 98%) was purchased for Kanto Chemical, Japan; and N-methylperfluorobutanesulfonamide (MeFBSA, > 97%) was purchased from Apollo Scientific. Milli-Q water was used throughout the experiments. LC–MS grade methanol and acetonitrile were acquired from Biosolve Chimie (Valkenswaard, The Netherlands). Ammonium acetate (≥ 99%) and glacial acetic acid (≥ 99%) were both purchased from Sigma-Aldrich, and ammonia solution (25%, analytical reagent grade) was acquired from Fisher.

### Sample preparation

Triplicate drinking water samples from Amsterdam (The Netherlands) were collected in 1-L HDPE bottles. The water samples were extracted then spiked with PFAS standards, and employed as the training dataset. Three different drinking water samples spiked with PFAS standard before extraction were extracted on different days to generate the test dataset.

The drinking water samples were extracted using solid phase extraction (SPE) as described elsewhere [34]. Briefly, the pH of all samples was adjusted to pH = 4 using acetic acid, then SPE was performed using Waters Oasis^®^ WAX SPE cartridges (3 mL, 60 mg, 30 μm). The SPE cartridges were preconditioned by passing a series of 3 mL 0.1% ammonium hydroxide in methanol, 3 mL of methanol, and then 3 mL of Milli-Q water. After loading the samples, the cartridges were washed with 3 mL ammonium acetate buffer solution (pH = 4). The cartridges were dried under high-purity nitrogen flow for 15 min. Next, the cartridges were eluted using 3 mL of 0.1% ammonium hydroxide in methanol. During elution, the extracts were filtered using FilterBio^®^ polypropylene (13 mm, 0.22 μm) syringe filters. The extracts were evaporated under a gentle stream of high-purity nitrogen to 75 µL, and then 175 µL of 0.05% acetic acid in water was added. The extracts were spiked with PFAS standards (5 µL, 0.2 ng/µL; in case of training set), then vortexed and centrifuged (5 min, 4000 RPM), after which they were transferred to LC vials for instrumental analysis.

Milli-Q water was extracted together with drinking water samples in triplicate as extraction blank.

### Sample analysis

All samples including blanks were analyzed by liquid chromatography (LC) coupled with HRMS. Aliquots of 10 μL were injected into Acquity UPLC CSH C18 column (130 Å, 2.1 × 150 mm, 1.7 μm). The mobile phase flow rate was set to 0.2 mL/min and the column temperature was set to 50 °C. The mobile phase consisted of 0.05% acetic acid in water (A) and 0.05% acetic acid in acetonitrile (B), and gradient elution was as described in Sadia et al. [35]. For HRMS, a MaXis 4 G high-resolution q-TOF-HRMS (Bruker, Leiderdorp, The Netherlands) with resolving power of 50,000 at m/z 300, and equipped with an ion-booster electrospray ionization (IB-ESI) source was employed. The mass spectra were recorded in negative mode with a mass range of 50–1500 m/z and a sampling rate of 5 Hz. To guarantee the required mass accuracy, internal mass calibration was carried out automatically for each analysis by infusing a 50 µM sodium acetate solution in a water–methanol mixture (1:1, v:v), with a loop injection of 20 μL at the beginning of the analysis (0.1–0.5 min).

### Studied algorithms

To assess our optimization approach, we employed five different algorithms, four of which are open-source and open-access. The used algorithms consisted of the SAFD, XCMS, OpenMS, KPIC2, and the DataAnalysis by Bruker. These algorithms represent a wide variety of feature detection strategies as well as underlying assumptions as briefly described below. Additionally, all algorithms were interfaced via patRoon.

#### SAFD

SAFD, an open-source algorithm, is a self-adjusting algorithm due to the fact that most of the parameters used in this algorithm are only the first guess and they get adjusted for each feature [31]. SAFD fits a pseudo-3D Gaussian function to the top 50% of the feature. This was also the only tested/investigated algorithm that is able to perform feature detection of both centroided and profile data via Cent2Prof algorithm [22], which predicts the mass peak width using the height, retention factor, and the m/z values. SAFD does not distinguish between potential adducts, isotopes, and/or in-source fragments and thus detects them as separate features.

#### XCMS

XCMS [20] is an open-source algorithm, which combines the regions of interest (ROI) detection in the mass domain and the centWave algorithm (CWA) for feature detection in the time domain [36, 37]. This combination has been one of the most commonly used approaches for the feature detection of LC-HRMS data [37, 38].

#### OpenMS

OpenMS [19] is an open-source software platform for processing of LC-HRMS data. OpenMS includes different algorithms for each step that takes place during NTS assays. One of such steps is feature detection, where a combination of feature detection and isotope detection is used for a robust and reliable feature detection. This algorithm first generates m/z traces based on the observed/measured mass error. Then these traces are fit with the FeatureFinderMetabo algorithm for the detection of the chromatographic peaks in the time domain. During the last step, only the features that fit the expected isotopic distribution are recorded as true features, while the others are considered as noise [39, 40]. The OpenMS algorithm has an isotope filtering step incorporated in its feature detection procedure, which is not included in other algorithms.

#### KPIC2

KPIC2 [21] is another open-source/access algorithm performing feature detection on centroided data. The feature detection method is based on pure ion chromatogram (PIC), by extracting the “pure ions” from the background noise, through tracking ions scan to scan and connecting data points with similar m/z values. This algorithm, similarly to the previous ones, generates RIO m/z traces in the mass domain and uses CWA to detect the features in the time domain. The main differentiating part of this algorithm is the use of k-means clustering to generate the ROIs rather than using the mass tolerances set by the user (e.g., XCMS and OpenMS).

#### Bruker DataAnalysis

Bruker DataAnalysis 4.4 (Bruker Daltonics) is a proprietary algorithm present in the Bruker software suite [41]. It can be used within the patRoon platform if the proprietary software is already installed and activated on the computer of interest. Bruker DataAnalysis uses the “Find Molecular Features” (FMF) algorithm for feature detection. This algorithm also operates using extracted ion chromatograms via centroided data. However, due to the nature of closed-source software, a detailed overview of how the algorithm operates cannot be provided. While automatic parameter control via patRoon for this algorithm is not feasible, manual adjustments can be made for each run individually using DataAnalysis.

### Experimental design and optimization

#### Parameter optimization

**Fig. 2:**
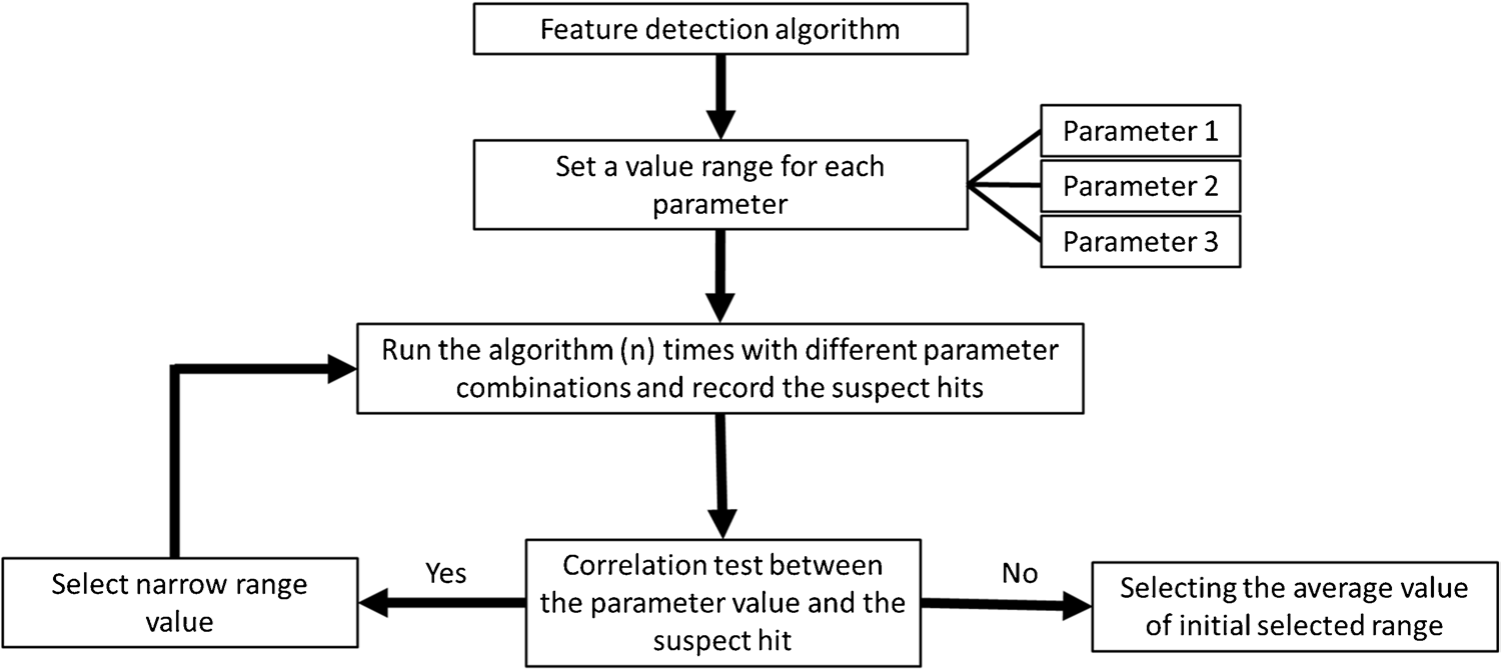
A schematic diagram of the optimization design used for optimizing feature detection algorithms

For the parameter optimization (Fig. 2, Table S2), we employed a stochastic strategy where, for each parameter, a range was defined based on a combination of extending the range of the default parameter and expert judgment. This ensured that the chosen ranges were both data-driven and informed by practical experience. Next, we randomly sampled a set of values from this range for each parameter and used these values for an iteration of feature detection followed by performing suspect screening (“Suspect screening” section). This process was repeated between 150 and 500 times depending on the algorithms’ computational intensity. In the next step, we evaluated the relationship between the number of detected suspects (which correspond to the true feature) vs each parameter. The parameters that showed a significant positive or negative linear correlation (i.e., *r* >|0.5| and *p* < 0.05) with the number of detected suspect analytes were considered for further optimization. For example, a parameter with an *r* value of − 0.6 indicated that a decrease in the parameter results in higher suspect hit rate and thus is a parameter to be optimized.

For the parameters that did not show any correlation with the number of suspect hits, the middle of the range was considered as the optimized value (Fig. 2). However, we acknowledge that this approach may overlook potential non-linear relationships. Non-linear optimization methods could be explored in future work to better account for such relationships.

It should be noted that this approach follows a Bayesian principal with an uninformative prior, implying that all values in the parameter space have the same probability of being the optimized value [42]. After the first sampling step, the prior probability distribution is updated based on observed correlation results. Specifically, parameters with strong correlations (either positive or negative) with the number of suspect hits help in identifying a narrower range of parameter values that are more likely to be optimal. This updated probability distribution, which now gives higher weights to these narrowed ranges, guides the subsequent sampling steps. This approach has the advantage of not having any initial assumptions while it suffers from the fact the optimization process may be computationally expensive. A list of parameters used for optimization for each algorithm is reported in Table S2 and the R script for parameter optimization found in the SI.

To compare our strategy with another existing algorithm for parameter optimization, we used the Isotopologue Parameter Optimization (IPO) algorithm [43], integrated within patRoon. The IPO automatically performs an evaluation of the sets of parameter values for an algorithm and selects the most optimized value by using natural, stable 13C isotopic peaks to calculate a peak picking score. A detailed overview of this method and the results can be seen in SI. It should be noted that IPO did not generate reasonable parameters. This limitation may stem from IPO’s dependence on specific sample characteristics; it was originally designed for metabolomic samples rather than environmental samples. Consequently, its results were not considered in our analysis.

#### Evaluation of algorithm performance

We tested two strategies to evaluate the algorithm optimization: false discovery rate (FDR) and suspect screening. These approaches were selected to accurately assess the quality of our optimization approach by defining the true positive feature using different methods. The FDR was employed to measure the effectiveness of the optimization procedure on structurally unknown features, while the suspect screening was used to evaluate the effectiveness of the optimization procedure on the known features that were previously spiked in the samples.

##### False discovery rate (FDR)

The FDR defines the number of cases wrongly identified as a true feature. The main difference between false positive rate (FPR) and FDR is the fact that for FPR the number of true negatives is necessary, which is not possible for HRMS data to extract without manual inspection of each feature. For the FDR, we used the equation below where FP is the number of false positives and TP is the number of true positives.

$$\text{FDR}\% = {\text{FP}}\,/\, {(\text{FP} + \text{TP})}$$

For the TPs, we made the assumption that features detected by multiple algorithms are more likely to be true peaks. Consequently, the features detected by only one algorithm were assumed FPs [32, 44]. This approach, overly simplistic method for defining the TPs and FPs and avoiding manual investigating of all data, has been used in various studies due to lack of alternatives [32, 44].

##### Suspect screening

To assess the effectiveness of our optimization strategy, we compared the number of detected suspects using default and optimized algorithm parameter settings. A suspect list of the 46 spiked PFAS standards was generated to screen for the TP features. This list included chemical identifiers (SMILES and InChiKeys), molecular formula, monoisotopic mass, adduct, the expected retention time, and at least one fragment for each suspect (Table S1).

For screening, the generated data at the MS1 level was screened against our suspect list using a m/z and retention tolerance of 5 mDa and 0.2 min, respectively. To confirm the detected suspect analytes, their MS2 signals were checked for the presence of potential fragments associated with our suspect analytes. The presence of at least one fragment was required to confirm the presence of a suspect analyte in our sample. This approach has been demonstrated as a robust strategy for suspect screening in complex environmental samples [9, 45, 46].

#### Alignment algorithm optimization

The OpenMS feature alignment algorithm [47] was selected for features alignment-and-grouping, due to its high performance. After optimizing the feature detection algorithms, the parameters of the alignment algorithm (Table S3) were subsequently optimized using the same approach described in the “Parameter optimization” section and Fig. 2, with 350 simulation runs.

All computations were performed on an 8-core, 16-thread CPU (Intel^®^ Core™ i7 10700) based PC with 32 GB of RAM, running Microsoft^®^ Windows^®^ 10 Education (64-bit). RStudio^®^ 2021.09.0 Build 351 was used to run patRoon 2.0.1 on this PC. Julia 1.7.2. was installed to be able to run SAFD.

In the context of this study, the term “number of features” refers to the number of all generated features derived from the analysis of both the water and blank samples. The concept of “feature groups” represents the number of feature group sets resulting from the alignment and grouping of features across all samples. The “filtered feature groups” referred to the feature group sets obtained after applying filtering criteria, as outlined in Table S4. The “suspect hits” referred to the number of suspects that were confirmed by exact mass, retention time, and the presence of a single fragment ion.

## Results and discussion

We performed feature detection on three drinking water extracts spiked with 46 PFAS standards as a training set, using five different algorithms. Parameter optimization (Table S5) was feasible for all open-source algorithms (SAFD, XCMS, OpenMS, KPIC2), while it was not possible for the remaining closed-source algorithm (Bruker DataAnalysis) due to its proprietary nature. We employed a combination of FDR and suspect screening to evaluate the applicability of our optimization approach. The training set was used for the parameter optimization, while the test set was employed for the final evaluation of the optimized parameters’ applicability to different samples.

### Feature detection algorithm parameters optimization

Under default parameter settings, the KPIC2 algorithm detected the largest number of features with around 150 k features, followed by XCMS and OpenMS with 19 k features each (Table 1). SAFD detected the smallest number of detected features, around 4 k. The filtering step led to a substantial reduction (average of 97%) in the number of feature groups for the optimized and default parameter settings. This reduction indicates that many detected features may have been noise or signals removed during blank subtraction (approximately 8% of feature groups were removed by blank subtraction).

For both the optimized and default parameters, detection rates of suspect analytes ranged from 14 positive hits for the Bruker software to 35 for OpenMS. After filtering, the detection ranged from 6 positive hits for Bruker to 31 for OpenMS (Table 1), representing a loss of 10% for suspect hit features for all algorithms except Bruker, which experienced a 57% loss. This loss was due to applied filtering criteria (Table S4) such as intensity threshold and blank subtraction. After optimization, OpenMS showed a substantial reduction of − 59% in features, from 18 to 7 K (Table 1). This optimization might have led to optimizing the filtering process and decreased in the detection of noise compared to the default settings, leading to a decline in both feature groups (− 54%) and suspect hits (− 9%).

Variations between default and optimized settings for XCMS and OpenMS were mainly caused by the signal-to-noise ratio (SNR) setting, while the other parameters had minimal influence on this variation (Figure S2, S3). Increasing SNR from 3 to 6 for OpenMS decreased features by − 59%, while decreasing SNR from 10 to 6 for XCMS increased features by + 11%, and that substantially influenced the number of feature groups (Table 1). SNR as a widely used technique in pre-preparation of the data file for the feature detection mainly removes the noise that potentially comes from instrument fluctuations [12]. However, SNR is fast and easy to perform, but it is unstable (very sensitive) and ignores the peak shape. Similarly, Dietrich et al. found that the intensity threshold and SNR are the parameters that significantly influence obtaining the false positive features [48].

During the initial optimization test for SAFD, only the SNR parameters showed a correlation (Figure S1). There was no difference between the default and the optimized setting for this parameter at the end of optimization. The rise in suspect hits and detected features in SAFD (Table 1, Fig. 4) was attributed to the increase in the number of iterations (maxNumbIter) (i.e., the number of performing all the steps in the algorithm for feature detection) from 1000 to 5000. This increases the TP features (e.g., suspect hits), but it generates more FP features (e.g., noise) that majority filtered by the filtering step (Table 1). Consequently, this resulted in longer execution time for feature detection, as indicated in Table 1. The increased iterations led to a higher number of detected features, subsequently elevating both TP features (e.g., suspect hits) and FP features.

**Table 1:** The number of features, feature groups, filtered groups, and suspect hits and the execution time for the studied algorithms using the default and optimized parameters

		SAFD		OpenMS		XCMS3		KIPAC2	Bruker DataAnalysis
		Default	Optimized (compared to default%)	Default	Optimized (compared to default%)	Default	Optimized (compared to default%)	Default	Default
Features		4391	17,218 (292%)	18,314	7465 (− 59%)	19,652	21,859 (11%)	151,108	17,781
Before filtering	Feature groups	1917	9417 (391%)	9097	4140 (− 54%)	10,131	12,519 (24%)	112,652	10,174
	Suspect hits	11	22 (100%)	35	32 (− 9%)	34	34 (0%)	30	14
After filtering	Feature groups	152	191 (26%)	169	116 (− 31%)	321	277 (− 14%)	277	181
	Suspect hits	11	20 (82%)	31	28 (− 10%)	28	32 (14%)	26	6
Execution time (min)		22.8	91.8 (303%)	1.7	0.9 (− 47%)	1.23	1.005 (− 18%)	154.8	2.2

The optimization approach did not sufficiently improve KIPC2 parameter settings due to a lack of correlation between parameter values and suspect hits (Figure S4). Bruker DataAnalysis, tested alongside open-source algorithms, was not possible to programmatically change due to the closed-source nature of the Bruker algorithm. Bruker DataAnalysis yielded the lowest suspect hits (6) among all algorithms tested after filtering (Table 1). This unexpected performance was particularly surprising given the expectation of good performance with data generated from Bruker instruments, which were used in this study. Moreover, DataAnalysis is an older software, and the newer Bruker software “MetaboScape” might offer improved performance. However, the use of default parameters resulted in suboptimal performance, emphasizing the need for manual optimization if feasible before its application. Hemmer et al. observed that the closed-source algorithm had been shown to generate a low number of true positive features as compared to the open-source algorithm [49].

OpenMS and XCMS demonstrated rapid execution, approximately 1 min, due to their use of mass traces for feature detection [20, 50, 51]. Conversely, KPIC2, employing k-means clustering for m/z value to find similarity, took 154.8 min, hindered by its incomplete use of ion intensity information [21]. This resulted in more low-intensity peaks and false positives, extending execution time. SAFD, using profile data, required 91 min, longer than OpenMS and XCMS, as it uses all data points in features, demanding more computational resources. Additionally, the HRMS data provided as centroid version and the need to convert centroided data to profile data increased execution time.

### Evaluation of algorithm performance

#### False discovery rate (FDR)

**Fig. 3:**
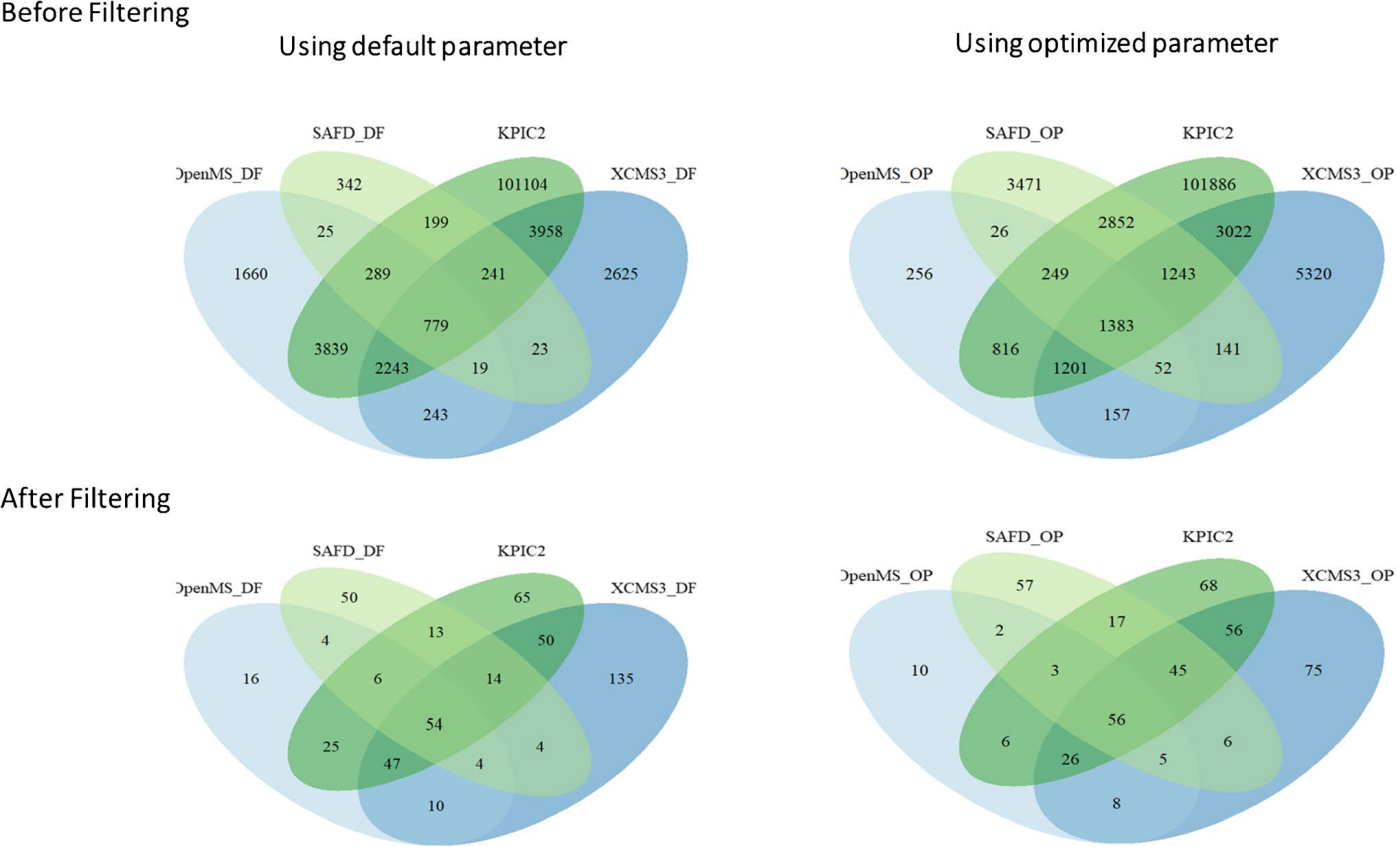
Venn diagram showing overlapping feature groups generated by the studied algorithms (OpenMS, SAFD, KPIC, and XCMS) using the default (DF) and optimized (OP) parameter settings. KPIC was not optimized so only default parameter was used in both cases

FDR calculations were based on both feature groups and filtered feature groups, employing three scenarios to define TP: first, the overlapping features between four algorithms (Fig. 3), second, the overlapping features between three algorithms (see Venn diagram Figure S9, S11 in the SI), and third, the overlapping features between two algorithms (see Venn diagram Figure S10, S12 in the SI).

After applying the filtering step in the case of optimized and default parameters, a decrease in the FDR was observed for all algorithms across all scenarios, except SAFD in the case of default parameters (Table 2). This reduction indicates the presence of a large number of FP feature groups generated from noise that was partially filtered out using the filtering criteria (Table S4).

In an ideal scenario, the algorithm’s performance should yield an FDR of 0%. However, this is not realistic for feature detection algorithms (Table 2), attributed to the high number of noise generated (FP) features. In the first and second scenarios, the optimized parameter settings led to an average 12% decrease in FDR% for all algorithms, except SAFD. This reduction in FDR% can be attributed to a decrease in the FP or increase in TP detection achieved through optimized parameter settings, this aligning with our approach of optimizing algorithms’ parameters to maximize suspect hits as TP features.

Conversely, in the case of SAFD, an increase in FDR% was observed after parameter settings optimization. This increase can be linked to a higher number of iterations, resulting in an increase in feature groups from 1917 with default parameters to 9417 with optimized parameters. This increase in iterations led to a higher count of FP, and this increased the chance of detecting both true peak and lesser quality peak (false peak) and getting a higher FDR after optimization.

In the third scenario, a different pattern was observed, with a high variation of FDR% ranging from 12 to 99% across all algorithms. While most cases showed a reduction in FDR% using optimized parameter settings, few cases get an increase in FDR%. This variation can be attributed to the TP assumption, wherein the overlap of two algorithms may not encompass all the TP features, and potentially leading to biased conclusions using the FDR approach.

**Table 2:** False discovery rate (FDR) calculated from feature groups and filtered feature groups using default (DF) and optimized (OP) parameter settings for studied algorithms, applying three scenarios of defining the true-positive (overlapped of 4 algorithms, overlapped of 3 algorithms, and overlapped of 2 algorithms)

Scenario to define the true-positive		Feature group							Filtered feature group						
		OpenMS		XCMS		SAFD		KPIC2	OpenMS		XCMS		SAFD		KPIC2
		DF	OP	DF	OP	DF	OP	DF	DF	OP	DF	OP	DF	OP	DF
Overlapping of 4 algorithms (scenario 1)		91%	67%	92%	89%	59%	85%	99%	66%	52%	82%	80%	63%	71%	79%
Overlapping of 3 algorithms (scenario 2)	KPIC2/SAFD/XCMS	-	-	90%	79%	46%	72%	99%	-	-	78%	63%	53%	46%	74%
	KPIC2/OpenMS/SAFD	88%	60%	-	-	44%	82%	99%	63%	48%	-	-	59%	69%	77%
	KPIC2/OpenMS/XCMS	67%	38%	70%	79%	-	-	97%	38%	29%	68%	70%	-	-	62%
	OpenMS/SAFD/XCMS	90%	62%	91%	87%	53%	83%	-	64%	47%	81%	78%	60%	68%	-
Overlapping of 2 algorithms (scenario 3)	OpenMS/XCMS	61%	29%	65%	77%	-	-	-	30%	18%	63%	66%	-	-	-
	OpenMS/SAFD	87%	53%	-	-	37%	79%	-	59%	43%	-	-	54%	65%	-
	OpenMS/KPIC2	21%	12%	-	-	-	-	94%	20%	22%	-	-	-	-	51%
	XCMS/SAFD	-	-	88%	75%	35%	67%	-	-	-	75%	60%	48%	41%	-
	XCMS/KPIC2	-	-	28%	45%	-	-	94%	-	-	47%	33%	-	-	39%
	SAFD/KPIC2	-	-	-	-	19%	38%	99%	-	-	-	-	40%	35%	67%

To validate our TP assumption, we randomly sampled features from feature groups and filtered feature groups from both overlapped and non-overlapped regions. The sample size was chosen to be more representative, at 10–20% of the overlapped region, to be 100 features for unfiltered feature groups and 10 features for filtered feature groups. Under the first scenario, our TP assumption held true, with all sampled overlapped filtered and non-filtered feature groups confirmed as true peaks (only 4 features out of 100 confirmed as noise), validating our initial hypothesis. Non-overlapped feature groups were identified as noise signals in feature groups, while filtered feature groups showed true peaks for an average of 50% sampled feature groups (Table S7).

In the second scenario, an average of 1% of sampled feature groups were confirmed as noise in the overlapped region in the filtered and non-filtered feature groups. The non-overlapped region in feature groups revealed an average of 4% sampled feature groups, while the filtered feature groups showed more true peaks in the non-overlapped region (Table S7). However, the third scenario yielded different results, with more noise signals detected in the overlapped region and a higher number of true peaks in the non-overlapped region compared to other scenarios. This discrepancy may explain the variation of FDR% (Table 2).

It is important to note that these scenarios do not confirm that all non-overlapped features were noise, as observed in the manual inspection of the sampling set in filtered feature groups, where true peaks were observed in all scenarios (refer to Table S7). Rather, it suggests that overlapped feature groups are more likely to be true peaks, as confirmed using the first and second scenarios. By increasing the number of overlapped algorithms, there is a greater chance of capturing more TP within this overlapped region. Employing optimized parameter settings for all algorithms increased the overlapped regains, representing an increase in TP features, as shown in case of first and second scenarios, except in one case (Venn diagrams in Fig. 3 and the SI). On the other hand, using the consensus of data from multiple algorithms is considered a useful approach to priorities the overlapped features as a true feature to be subsequently used in the NTS.

#### Suspect screening

**Fig. 4:**
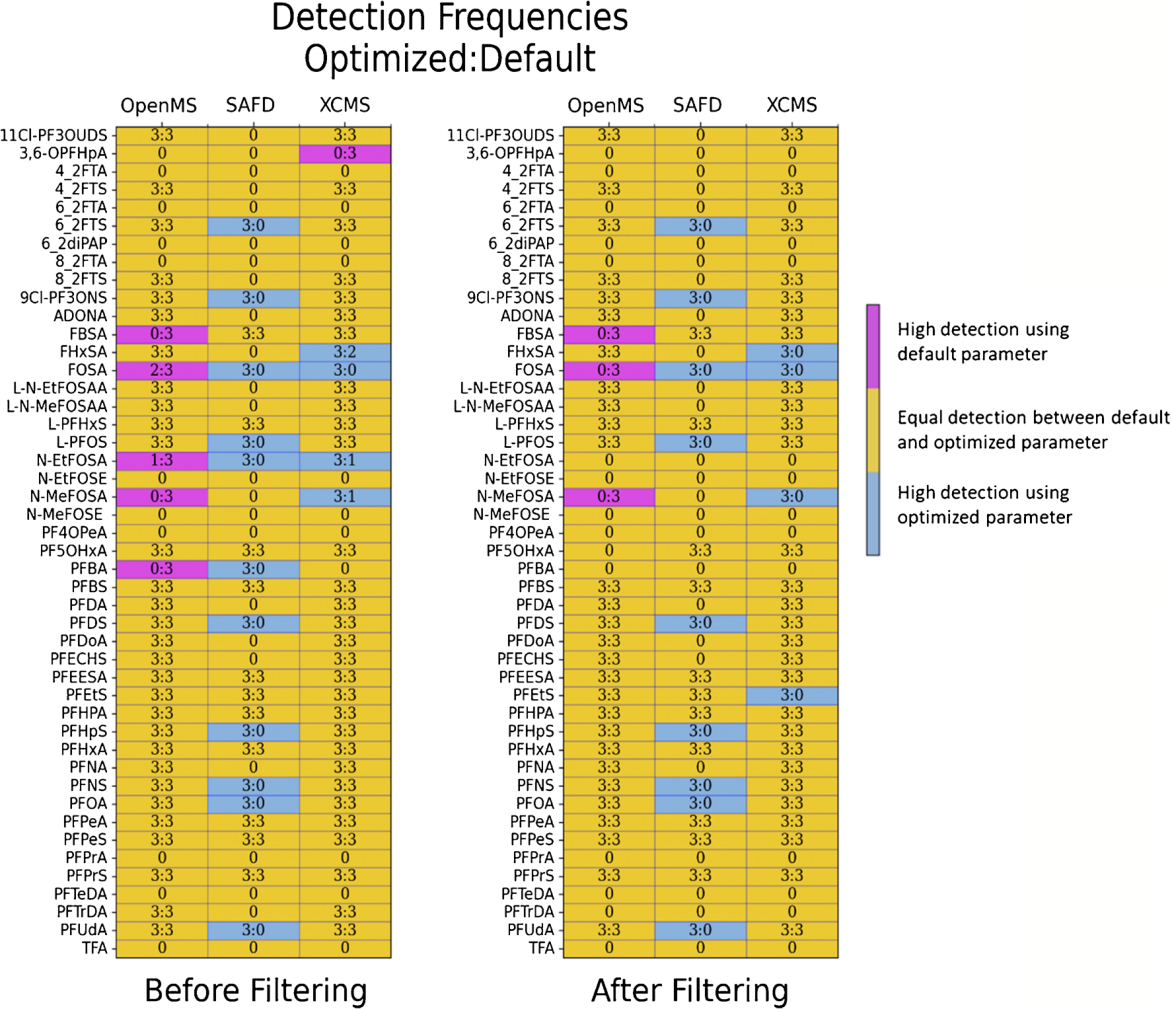
The frequent detection for each individual suspect in the triplicate samples (training set) using optimized and default parameter settings for each algorithm (OpenMS, SAFD, and XCMS), before and after applying the filtering step in the NTS

The parameter optimization consistently improved the detection frequencies of the suspect analytes across all algorithms, except for OpenMS (Fig. 4, Table 1). The algorithm which is the most impacted by the optimization was SAFD with doubling the number of the suspect hits after optimization.

Through an examination of frequent detection patterns for individual PFAS in the suspect list (Fig. 4), noteworthy observations arise. It can be seen that certain features were detected inconsistently across triplicate samples (e.g., FOSA), while others were exclusively detected using the default algorithm and not in the optimized version (e.g., 3,6-OPFHpA, FBSA). On the contrary, several suspects showed detectability across all algorithms employed (e.g., PFEESA, PFBS, 4_2FTS), whereas other suspects were not detected across all algorithms (e.g., 4_2FTS, 8_2FTS, ADONA), or were not detected by any algorithms (e.g., 4_2FTA, 8_2FTA, PF4OPeA).

To explain the lack of congruences in feature detection between different algorithms and between default and optimized parameter settings, further manual investigations of the peak shape and intensity signals using proprietary software (DataAnalysis) were performed. In some cases, low peak intensity (1 × 10^3^), almost near to the noise signal, which made it challenging for the algorithm to distinguish between the signal and background instrumental noise, resulting in non-detection by the studied algorithm (e.g., 4_2FTA, PF4OPeA, PFPrA, TFA). In other cases, the features were with low intensity (10^3^) and detected by the employed algorithm. Still, features were filtered out in the filtering step (e.g., 3.6-OPFHpA, N-EtFOSA, PFBA), due to filtering criteria (Table S4).

**Fig. 5:**
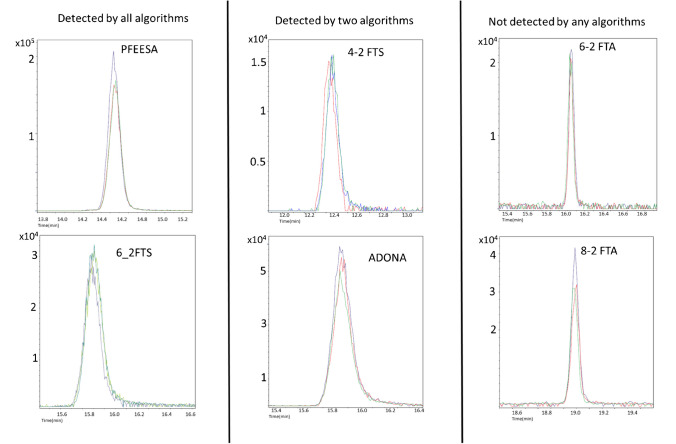
Peak chromatogram extracted using DataAnalysis software for selected suspect chemicals, including different scenarios of detection by the studied algorithms

Conversely, two suspects (6_2FTA, 8_2FTA) were not detected despite their high intensities (10^4^) and well-defined Gaussian shapes. This lack of detection could be attributed to the uniquely narrow peaks exhibited by these suspects (as shown in Fig. 5). The narrow peaks provide fewer data points for the algorithms to extract from the mass domain, making it more challenging for the algorithms to identify them as proper chromatographic peaks after extracting the m/z values from the raw data (see the peak chromatogram in Fig. 5).

By using both evaluation steps (FDR and suspect screening) for feature detection algorithms, it can be shown that the use of optimized parameters improves the performance of the algorithms. This improvement is evidenced by either an increase in TPs features, as demonstrated by the increased suspect hits in the suspect screening, or a reduction in FPs features, as indicated by a decrease in the FDR%.

While Fig. 4 suggests that default and optimized parameters often yield similar results, it was clear in a specific scenario where parameter optimization significantly impacts the results. For instance, the optimization led to a doubling of the suspect hits for the SAFD algorithm, illustrating a clear benefit. Additionally, the reduction in FDR% in most scenarios with optimized parameters (Table 2) indicates that optimization helps in reducing the number of FPs, particularly in complex datasets where noise can generate numerous false detections. For instance, the optimization led to 24% reduction in FDR% for OpenMS. The optimized parameters help filter out such noise, leading to a more accurate identification of true features.

It is important to note that the default algorithm parameters in patRoon were changed to suit the data generated for our instrument (patRoon developed in our institute). This pre-optimization may contribute to the smaller observed differences between default and optimized settings in some cases. Additionally, the inherent robustness of some algorithms to parameter changes or the nature of the dataset itself may also play a role. For example, certain PFAS compounds with low peak intensity or narrow peaks may still pose detection challenges, even with optimized parameters.

### Alignment and grouping algorithm optimization

Applying the same optimization approach used for feature detection to the alignment-and-grouping algorithm revealed no significant correlation between the parameter values (Table S3) and suspect hits in this context.

For feature alignment-and-grouping, only the maxGroupRT parameter demonstrated a moderate correlation (*R* = 0.62) with suspect hits when using features generated from OpenMS, SAFD, and XCMS (Figure S5, S6, S7). However, no correlation was observed when using features generated via KPIC2 (Figure S8). The maxGroupMZ parameter showed no correlation with suspect hits across all feature detection algorithms. The correlation plots displayed a scattered pattern (see the correlation plot in the Supplementary Information), indicating that the algorithm tends to yield artifact inconsistent results, regardless of the input data. The dataset in our case was limited to three samples of a drinking water matrix measured in the same batch, resulting in minimal variation due to retention time drift and matrix effects. Consequently, the algorithm demonstrated sufficient performance regardless of parameter changes. In scenarios involving larger sample batches and more complex matrices, such as wastewater, optimization might prove crucial, potentially revealing differences between default and optimized settings.

### Applicability of the optimization approach

**Fig. 6:**
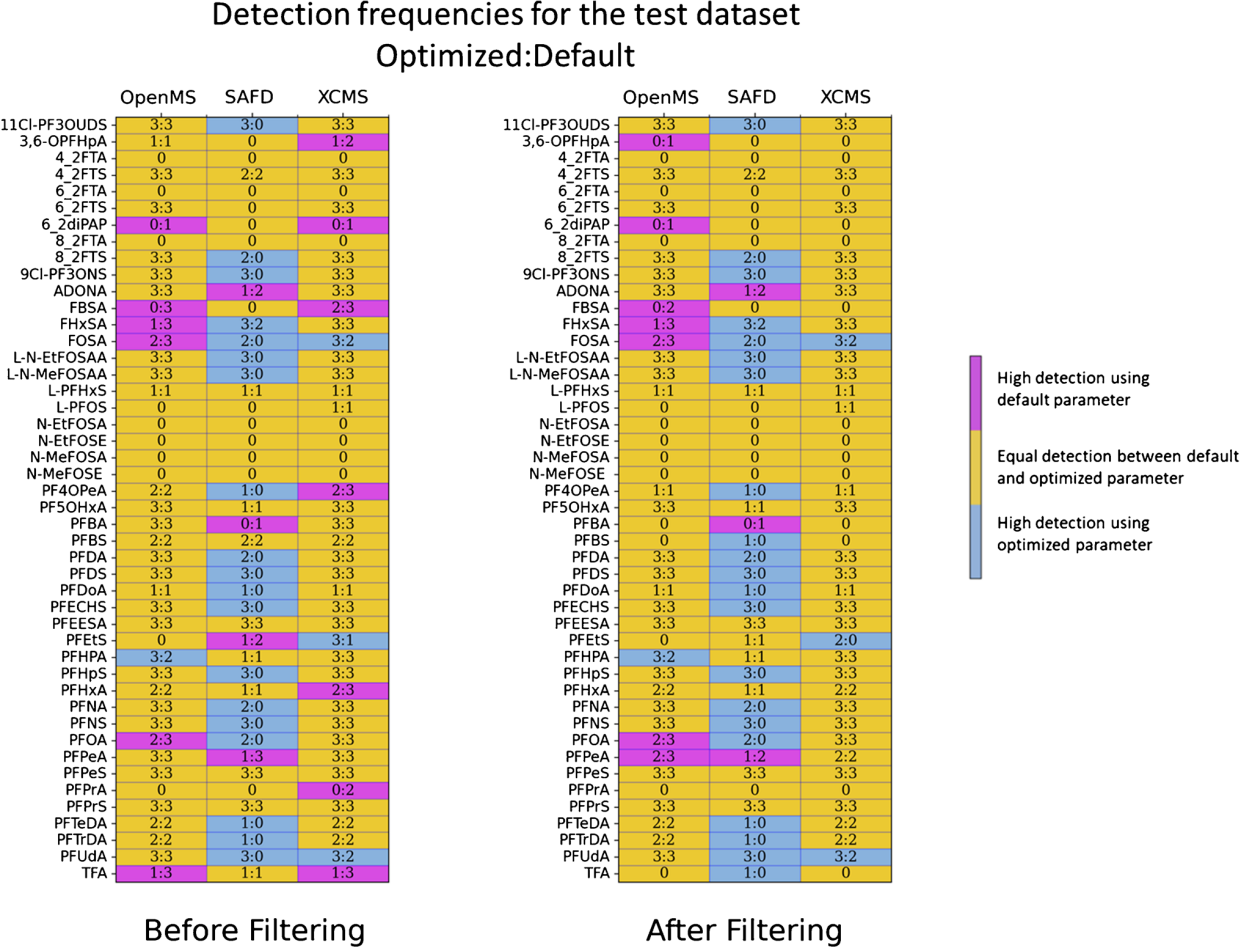
The frequent detection for each suspect in the three drinking water samples (test dataset) using optimized and default parameter for each algorithm (OpenMS, SAFD, and XCMS)

Using both optimized and default settings of the studied algorithms on the test dataset produced comparable results to those on the training dataset (Fig. 6, Table S6). This improvement in feature detection was evident, either through an increase in the number of suspect hits in the case of SAFD and XCMS, or by a reduction in the number of feature groups, mainly FP features, for OpenMS (Table S6). These findings indicate that our optimization approach successfully enhanced feature detection on the test set by optimizing algorithm parameters using the training set, showing promise for future research in this area.

In the context of NTS, achieving optimal performance in feature detection requires a careful balance between maximizing the number of detected features and minimizing the FDR% (increasing the TPs and decreasing the FP). Although it may seem intuitive that an algorithm yielding a higher number of TPs with a low FDR% would perform better. It is important to note that the efficacy of feature detection can be dependent on the sample characteristics, such as matrix composition and targeted chemical classes (Rafiei and Sleno, 2015).

The selection of algorithms should be guided by the specific application’s requirements and user needs, whether it necessitates an inclusive approach, as demonstrated by KPIC2 with a high number features and consequently a high number of FP features, or a more selective approach, as demonstrated by SAFD, which generates fewer features of higher quality but risks missing some TP features.

Thus, it is recommended to perform parameter optimization early in the NTS workflow regardless of the chosen algorithm, preferably using a sample spiked with the target group of chemicals to fine-tune algorithm parameters. This optimization approach aims to maximize the detection of TP features (e.g., suspect hits). In our study, focusing on drinking water samples and targeting PFAS as the chemical class, we used a PFAS list for parameter optimization. While this strategy does not guarantee the detection of all TP peaks or the elimination of all FP, it effectively mitigates FP by enhancing TP peak detection, thereby reducing the complexity of NTS analysis.

However, there are several limitations to our method that should be considered. Firstly, the current optimization approach does not account for parameter interactions, which can significantly impact the performance of feature detection algorithms. Future work could expand the optimization strategy to consider these interactions, potentially through multifactorial experimental designs or advanced stochastic optimization methods. Additionally, the computational intensity of the optimization approach could be a limiting factor in some applications, as the process may require substantial computational resources, particularly for complex or large datasets.

Furthermore, the efficacy of the method may be dependent on specific sample characteristics, such as matrix composition and the nature of the targeted chemical classes. This dependency can affect the generalizability of the optimization results to different sample types or analytical contexts. As a result, it is essential to validate the optimization process with various sample matrices to ensure its robustness and applicability across different scenarios.

By addressing these limitations and refining the optimization process, we can further improve the reliability and accuracy of feature detection in NTS. Despite these limitations, our approach offers advantages over using the default parameters. The proposed method improved the performance of feature detection algorithms, which can lead to increase the detection of TP features, and is going to be integrated in the future version of patRoon. By addressing the computational and interaction-based limitations in future work, our approach has the potential to become even more robust and widely applicable.

## Conclusion

Feature detection algorithms require careful selection of algorithm parameters due to the importance of reliable data for the subsequent steps in the NTS. However, selecting appropriate algorithm parameters is complex, and manual fine-tuning can lead to unpredictable data quality outcomes. To address this challenge, we developed a novel optimization method within the patRoon platform to automate the fine-tuning of parameter settings of feature detection algorithms.

Our study demonstrates that using our approach for parameter optimization enhances the performance of feature detection algorithms. This optimization results in improved detection frequencies of suspect analytes and reductions in FDR%. Despite variations in outcomes observed with different algorithms, optimizing parameters reduces the risk of losing true peaks from the original data.

**Fig. 7:**
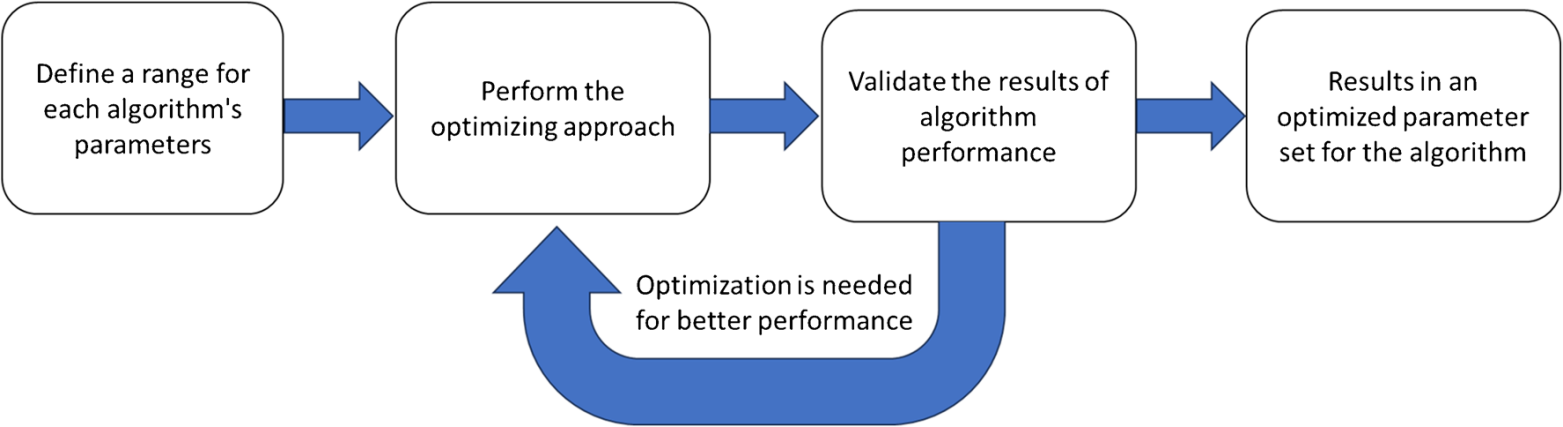
A schematic diagram for the future application of the optimization approach

To apply this optimization strategy with other algorithms, one should define parameter ranges, perform random sampling and iterations, evaluate performance, optimize parameters based on significant correlations, and validate the results (Fig. 7). We plan to integrate our optimization approach into future versions of patRoon to facilitate its application and improve feature detection performance. Employing multiple feature detection workflows can significantly enhance TP detection, as overlapping features identified by different algorithms are more likely to be TPs. This approach leverages the strengths of each algorithm, reducing the likelihood of missing true features due to algorithm-specific biases or limitations. As observed in our work, the overlapping regions between different algorithms serve as valuable indicators TP features and this could be used for the prioritization step in the NTS.

However, ranking the performance of the algorithms may not be meaningful, as each algorithm operates differently and requires optimization adopted to specific research objectives, sample matrices, and instrument configurations. Therefore, algorithm selection should align closely with the unique requirements of the application.

Our study underscores the importance of early parameter optimization in the NTS workflow to maximize the detection of true positive features, simplifying subsequent analyses and reducing complexity. By optimizing algorithms to maximize suspect hits as TP features in samples spiked with chemical structurally similarly of the interested chemical classes, this approach would increase the detection of TP features. Future research can explore more refined methods for increasing TPs and reducing the FP features to further enhance the accuracy of NTS analyses.

## Supplementary Information

Below is the link to the electronic supplementary material.

Supplementary file1 (DOCX 806 KB)

Supplementary file2 (TXT 18 KB)
