# Supplementary material for: A stochastic approach for parameter optimization of feature detection algorithms for non-target screening in mass spectrometry
Source: Anal Bioanal Chem. 2024 Jul 12;417(27):6033–47. doi: 10.1007/s00216-024-05425-3 (PMC12583407; doi:10.1007/s00216-024-05425-3)
Supplement: Supplementary file 1 — Supplementary file1 (DOCX 806 KB) [file 216_2024_5425_MOESM1_ESM.docx]

Supplementary Information

**A stochastic approach for parameter optimization of feature detection algorithms for non-target screening in mass spectrometry**

Mohammad Sadia^a^, Youssef Boudguiyer^a^, Rick Helmus^a^, Marianne Seijo^a^, Antonia Praetorius^a^, Saer Samanipour^b^.

^a^ Institute for Biodiversity and Ecosystem Dynamics, University of Amsterdam, Amsterdam, The Netherlands.

^b^ Van ‘t Hoff Institute for Molecular Sciences (HIMS), University of Amsterdam, Amsterdam, The Netherlands

# Isotopologue Parameter Optimization

The Isotopologue Parameter Optimization (IPO) was developed to automatically optimize the parameter settings of XCMS algorithm for metabolomics analysis [1]. It uses natural stable ^13^C isotopes to calculate a peak picking score (PPS). Users can define an initial parameter range for optimization, and IPO maximizes its PPS, which in essence means that the algorithm attempts to increase the number of detectable features. We applied IPO to optimize the parameter of other tested algorithms using environmental datasets (in our case drinking water).

Initially, OpenMS and XCMS3 were optimized by applying the IPO algorithm optimization, due to their fast feature detection. A large number of features for both OpenMS (~ 3 million features) and XCMS3 (88750 features) were generated by using the IPO optimization. However, some parameters, like noiseThrInt, chromSNR, chromFWHM, minFWHM for OpenMS, and snthresh and noise for XCMS3, exceeded their lowest possible value and iterated down to a value of one, leading to unrealistic settings, which might be an error that limit our test. On the contrary, IPO had shown to be valuable in selecting appropriate parameters for metabolomics data analysis [1, 2]. The IPO algorithm optimizes feature detection algorithms by trying to maximize the number of features that could be detected with high PPS. As a result, it will seek as many features as possible, leading to iterating the parameter values downwards as this yields more features. These features however are probably mostly background noise. Due to this limitation, we did not further test IPO with other algorithms.

Table S1: List of PFAS in the spiked mixture.

| *Compound* | *Acronym* | *Molecular Formula* | *MS2_Fragment* | *Retention time (s)* |
| --- | --- | --- | --- | --- |
| Trifluoroacetic acid | TFA | C2HF3O2 | CF3 | 272 |
| Pentafluoropropionic acid | PFPrA | C3HF5O2 | C2F5 | 434 |
| Perfluoropropanesulfonic acid | PFPrS | C3HF7O3S | SO3 | 780 |
| Potassium pentafluoroethanesulfonate | PFEtS | C2HF5O3S | SO3; FSO3 | 570 |
| Perfluorobutyric acid | PFBA | C4HF7O2Ê | C3F7 | 611 |
| Perfluoropentanoic acid | PFPeA | C5HF9O2 | C4F9 | 778 |
| Perfluorohexanoic acid | PFHxA | C6HF11O2 | C5F11 | 919 |
| Perfluoroheptanoic acid | PFHPA | C7HF13O2 | C6F13 | 1050 |
| Perfluorooctanoic acid | PFOA | C8HF15O2 | C8F15O2 | 1167 |
| Perfluorononanoic acid | PFNA | C9HF17O2 | C8F17 | 1279 |
| Perfluorodecanoic acid | PFDA | C10HF19O2 | C9F19 | 1369 |
| Perfluoroundecanoic acid | PFUdA | C11HF21O2 | C10F21 | 1437 |
| Perfluorododecanoic acid | PFDoA | C12HF23O2 | C12F23O2 | 1487 |
| Perfluorotridecanoic acid | PFTrDA | C13HF25O2 | C13F25O2 | 1526 |
| Perfluorotetradecanoic acid | PFTeDA | C14HF27O2 | C14F27O2 | 1554 |
| Perfluorobutylsulphonamide | FBSA | C4H2F9NO2S | NO2S | 794 |
| Perfluorohexanesulfonamide | FHxSA | C6H2F13NO2S | NO2S | 990 |
| Perfluorooctanesulfonamide | FOSA | C8H2F17NO2S | NO2S | 1147 |
| N-methylperfluorooctane sulfonamidoacetic acid | L-N-MeFOSAA | C11H6F17NO4S | C8F17 | 1279 |
| N-ethylperfluorooctane sulfonamidoacetic acid | L-N-EtFOSAA | C12H8F17NO4S | C8F17 | 1311 |
| Potassium perfluoro-1-butanesulfonate | PFBS | C4HF9O3S | FSO3;SO3 | 929 |
| Sodium perfluoro-1-pentanesulfonate | PFPeS | C5HF11O3S | FSO3;SO3 | 1060 |
| Potassium perfluorohexanesulfonate | L-PFHxS | C6HF13O3S | FSO3;SO3 | 1172 |
| Sodium perfluoro-1-heptanesulfonate | PFHpS | C7HF15O3S | FSO3;SO3 | 1267 |
| Potassium perfluorooctanesulfonate | L-PFOS | C8HF17O3S | FSO3;SO3 | 1344 |
| Sodium perfluoro-1-nonanesulfonate | PFNS | C9HF19O3S | FSO3;SO3 | 1410 |
| Sodium perfluoro-1-decanesulfonate | PFDS | C10HF21O3S | FSO3;SO3 | 1456 |
| Sodium 1H, 1 H,2H,2H-perfluoro-1-hexanesulfonate | 4_2FTS | C6H5F9O3S | C6H3F8SO3;SO3 | 832 |
| Sodium 1 H, 1 H,2H,2H-perfluoro-1-octanesulfonate | 6_2FTS | 6-2FTS | C8H3F12SO3;SO3 | 1082 |
| Sodium 1 H, 1 H,2H,2H-perfluoro-1-decanesulfonate | 8_2FTS | C10H5F17O3SÊ | C10H3F16SO3;SO3 | 1298 |
| Sodium dodecafluoro-3H-4,8-dioxanonanoate | ADONA | C7H2F12O4 | C4F9O2 | 1078 |
| Potassium 9-chlorohexadecafluoro-3-oxanonane-1-sulfonate | 9Cl-PF3ONS | C8HClF16O4S | C8F12ClO | 1396 |
| Potassium 11-chloroeicosafluoro-3-oxaundecane-1-sulfonate | 11Cl-PF3OUDS | C10ClF20HO4S | C8F20ClO | 1482 |
| Perfluoro-4-oxapentanoic acid | PF4OPeA | C4HF7O3 | CF3O | 680 |
| Perfluro-5-oxahexanoic acid | PF5OHxA | C5HF9O3 | CF3O | 822 |
| Perfluoro-3,6-dioxaheptanoic acid | 3,6-OPFHpA | C5HF9O4 | CF3O | 899 |
| Potassium perfluoro(2-ethoxyethane)sulfonate | PFEESA | C4F9HO4S | CF3O | 994 |
| Potassium perfluoro-4-ethylcyclohexanesulfonate | PFECHS | C8HF15O3S | C8F15;FSO3 | 1266 |
| Sodium bis(1H,1H,2H,2H-perfluorooctyl)phosphate | 6_2diPAP | C16H9F26O4P | H2O4P | 1572 |
| N-ethylperfluoro-1-octanesulfonamidoacetic acid | N-EtFOSA | C10H6F17NO2S | C3F7 | 1306 |
| N-methylperfluoro-1-octanesulfonamidoacetic acid | N-MeFOSA | C9H4F17NO2S | C3F7 | 1257 |
| 2-(N-ethylperfluoro-1-octanesulfonamido)-ethanol | N-EtFOSE | C12H10F17NO3S | C2H3O2 | 1306 |
| 2-(N-methylperfluoro-1-octanesulfonamido)-ethanol | N-MeFOSE | C11H8F17NO3S | C2H3O2 | 1257 |
| 3-Perfluoropropyl propanoic acid (3:3) | 4_2FTA | C6H5F7O2 | C5H3F6 | 753 |
| 3-Perfluoropentyl propanoic acid (5:3) | 6_2FTA | C8H5F11O2 | C7HF8;C7F7 | 976 |
| 3-Perfluoroheptyl propanoic acid (7:3) | 8_2FTA | C10H5F15O2 | C9HF12 | 968 |

**Table S2.** Parameters used for optimization of each feature detection algorithm along with their descriptions.

| Algorithm | Parameter | Description |
| --- | --- | --- |
| SAFD[3] | Resolution | the expected mass resolution of the used instrument |
|  | minInt | the minimum signal intensity for the features |
|  | maxNumbIter | the maximum number of cycles that the algorithm will perform |
|  | minPeakWS | removes features that have less seconds than the threshold |
|  | maxTPeakW | removes features that have more seconds than this threshold |
|  | S2N | the signal-to-noise ratio threshold. |
| OpenMS[4] | noiseThrInt | sets the noise intensity threshold |
|  | chromSNR | sets the signal-to-noise intensity threshold |
|  | chromFWHM | sets the expected chromatographic peak width denoted in seconds |
|  | minFWHM | the minimum FWHM a feature must have denoted in seconds |
|  | maxFWHM | sets the maximum FWHM for a feature denoted in seconds |
|  | mzPPM | sets the maximum allowed mass deviation within a mass trace |
| XCMS[5] | snthresh | sets the signal-to-noise ratio threshold |
|  | peakwidthmin | sets the expected minimum peak width |
|  | peakwidthmax | sets the expected maximum peak width |
|  | mzPPM | sets the maximum m/z tolerance within a mass trace |
|  | k | sets a minimum threshold of how many peaks should be present within a mass trace |
|  | minIntensity | sets a minimum threshold of how many peaks should be present within a mass trace |
|  | noise | essentially performs the same as the minIntensity parameter. |
| KPIC2[6] | level | only retains mass traces if their maximum values are over a certain threshold |
|  | min_snr | sets the minimal signal-to-noise ratio |

**Table S3.** Parameters used for optimization of the OpenMS alignment-and-grouping algorithm along with their descriptions.

| Alignment algorithm | **Parameter** | **Description** |
| --- | --- | --- |
| OpenMS - feature alignment | maxAlignRT | sets the maximum retention time |
|  | maxAlignMZ | sets the maximum m/z difference |
| OpenMS - feature grouping | maxGroupRT | sets the maximum retention time difference between features |
|  | maxGroupMZ | sets the maximum m/z difference limit. |

**Table S4.** Parameter used in filtering step along with their descriptions

| Filter | Value | Description | |  |
| --- | --- | --- | --- | --- |
| absMinIntensity | 10000 | | Minimum intensity in absolute terms for features to be kept. | |
| preAbsMinIntensity | 100 | | Identical to absMinIntensity but applied before any other filtering step. | |
| relMinReplicateAbundance | 1 | | Minimum relative abundance of a feature that must be present within a feature group. | |
| maxReplicateIntRSD | 0.75 | | Maximum relative standard deviation (RSD) of intensities for features within a feature group. A feature group is removed if the RSD is above this threshold. | |
| blankThreshold | 5 | | Feature groups that are also present in the blanks are removed unless their relative intensity is above this value. | |
| retentionRange | 200-inf | | Range of retention time (in seconds) | |
| mzRange | 100-1000 | | Range of mass (in m/z) | |

**Table S5.** Selected parameters and values used for optimizing feature detection parameters.

| Algorithm | Parameters | First optimization round | | Second optimization round | | Third optimization round | | Optimized values | | Default | |  |
| --- | --- | --- | --- | --- | --- | --- | --- | --- | --- | --- | --- | --- |
|  |  | **Selected initial values** | **Correlation** | **New values** | **Correlation** | **New values** | **Correlation** |  |  |  |  |  |
| SAFD | Resolution | 10^4^ - 10^5^ | No | 50000 | - | - | | 50000 | | 30000 | |  |
|  | minInt | 10^3^-5000 | No | 3000 | - |  |  | 3000 | | 2000 | |  |
|  | maxNumbIter | 1000-5000 | No | 3000 | - |  |  | 5000 | | 1000 | |  |
|  | minPeakWS | 3—10 | No | 6 | - |  |  | 6 | | 3 | |  |
|  | maxTPeakW | 11—30 | No | 20 | - |  |  | 20 | | 300 | |  |
|  | S2N | 3-100 | Yes (-0.65) | 2—10 | No |  |  | 2 | | 2 | |  |
| OpenMS | noiseThrInt | 1000-5000 | Yes (-0.74) | 500-3000 | Yes (-0.82) | 500-1000 | No | 750 | | 1000 | |  |
|  | chromSNR | 3-100 | Yes (-0.43) | 2—10 | No | 6 | - | 6 | | 3 | |  |
|  | chromFWHM | 3—30 | No | 15 | - | 15 | - | 15 | | 5 | |  |
|  | minFWHM | 3—10 | No | 6 | - | 6 | - | 6 | | 1 | |  |
|  | maxFWHM | 11—30 | No | 20 | - | 20 | - | 20 | | 30 | |  |
|  | mzPPM | 2—30 | No | 15 | - | 15 | - | 15 | | 10 | |  |
| XCMS3 | snthresh | 2-100 | Yes (-0.6) | 44836 | No | - | | 6 | | 10 | |  |
|  | peakwidthmin | 3—10 | No | 6 | - |  |  | 6 | | 20 | |  |
|  | peakwidthmax | 11—30 | No | 20 | - |  |  | 20 | | 50 | |  |
|  | mzPPM | 2—30 | No | 15 | - |  |  | 15 | | 25 | |  |
|  | k | 2—10 | No | 6 | - |  |  | 6 | | 3 | |  |
|  | minIntensity | 1000-5000 | No | 3000 | - |  |  | 3000 | | 1000 | |  |
|  | noise | 1000-5000 | No | 3000 |  |  |  | 3000 | | 0 | |  |
| KPIC2 | level | 1000-5000 | No | - | | | | | 1000 | |  | |
|  | min_snr | 3-100 | No |  |  |  |  |  | 4 | |  | |

**Table S6 :** The number of features, feature groups, filtered groups, and suspect hits for the studied algorithms using the default and optimized parameters for the test dataset.

|  |  | SAFD | | OpenMS | | XCMS3 | | KPIC2 | Bruker |
| --- | --- | --- | --- | --- | --- | --- | --- | --- | --- |
|  |  | Default | Optimized  (compare to default%) | Default | Optimized  (compare to default%) | Default | Optimized  (compare to default%) | Default | Default |
| Features | Sample1 | 1401 | 2612 (86%) | 6723 | 2909 (-57%) | 8563 | 7116 (-17%) | 59070 | 6537 |
|  | Sample2 | 1360 | 2526 (86%) | 6112 | 2625 (-57%) | 8967 | 6598 (-26%) | 55127 | 5922 |
|  | Sample3 | 1367 | 2222 (63%) | 7685 | 3152 (-59%) | 8371 | 7576 (-9%) | 59320 | 7261 |
| Feature groups | Sample1 | 1151 | 2185 (90%) | 5536 | 2433 (-56%) | 7055 | 6060 (-14%) | 55093 | 5542 |
|  | Sample2 | 1130 | 2198 (95%) | 5141 | 2260 (-56%) | 7734 | 5822 (-25%) | 51950 | 5133 |
|  | Sample3 | 1193 | 2006 (68%) | 6843 | 2937 (-57%) | 7399 | 6931 (-6%) | 56050 | 6668 |
| Suspect hits | Sample 1 | 9 | 25 (178%) | 30 | 30 (0%) | 31 | 32 (3%) | 28 | 10 |
|  | Sample 2 | 8 | 23 (188%) | 32 | 27 (-16%) | 33 | 30 (-9%) | 32 | 7 |
|  | Sample 3 | 11 | 21 (91%) | 32 | 28 (-13%) | 36 | 33 (-8%) | 33 | 11 |
| Filtered feature groups | Sample1 | 406 | 721 (78%) | 2563 | 489 (-81%) | 899 | 1463 (63%) | 1351 | 849 |
|  | Sample2 | 365 | 709 (94%) | 2156 | 406 (-81%) | 864 | 1404 (63%) | 1194 | 667 |
|  | Sample3 | 659 | 1354 (105%) | 1536 | 1029 (-33%) | 1755 | 2836 (62%) | 2770 | 1883 |
| Filtered suspect hits | Sample1 | 7 | 20 (186%) | 27 | 26 (-4%) | 25 | 27 (8%) | 25 | 5 |
|  | Sample2 | 7 | 23 (229%) | 30 | 25 (-17%) | 26 | 28 (8%) | 27 | 4 |
|  | Sample3 | 9 | 21 (133%) | 27 | 25 (-7%) | 28 | 28 (0%) | 28 | 8 |

**Table S7.** Results from manual inspection of sampled 100 features from feature groups and 10 features from filtered feature groups, respectively. The table indicates the count of noise in the overlapped regions and the number of true peaks in the non-overlapped regions for both feature groups and filtered feature groups.

| Scenario to define the true-positive | | Feature group (sampling of 100 features) | | | | Filtered feature group (sampling of 10 features) | | | |
| --- | --- | --- | --- | --- | --- | --- | --- | --- | --- |
|  |  | DF | | OP | | DF | | OP | |
|  |  | noise (overlapped region) | peak (non-ovelapped regoin) | noise (overlapped region) | peak (non-ovelapped regoin) | noise (overlapped region) | peak (non-ovelapped regoin) | noise (overlapped region) | peak (non-ovelapped regoin) |
|  |  |  |  |  |  |  |  |  |  |
|  |  |  |  |  |  |  |  |  |  |
| Overlapping of 4 algorithms | | 3 | 2 | 4 | 2 | 0 | 4 | 0 | 6 |
| Overlapping of 3 Algorithms | KPIC2/SAFD/XCMS | 2 | 4 | 3 | 0 | 2 | 4 | 1 | 7 |
|  | KPIC2/OpenMS/ SAFD | 4 | 1 | 5 | 0 | 0 | 5 | 2 | 4 |
|  | KPIC2/OpenMS/XCMS | 6 | 3 | 6 | 1 | 0 | 5 | 0 | 4 |
|  | OpenMS/SAFD/XCMS | 2 | 6 | 4 | 4 | 0 | 6 | 0 | 5 |
| Overlapping of 2 Algorithms | OpenMS/XCMS | 45 | 22 | 30 | 12 | 2 | 2 | 2 | 6 |
|  | OpenMS/SAFD | 8 | 40 | 16 | 31 | 2 | 5 | 1 | 5 |
|  | OpenMS/KPIC2 | 44 | 4 | 32 | 2 | 1 | 4 | 1 | 4 |
|  | XCMS/SAFD | 12 | 20 | 6 | 25 | 0 | 3 | 0 | 6 |
|  | XCMS/KPIC2 | 48 | 8 | 45 | 4 | 3 | 3 | 1 | 4 |
|  | SAFD/KPIC2 | 8 | 4 | 23 | 4 | 0 | 4 | 1 | 6 |

# Correlation plots of feature detection algorithms

| SAFD - First optimization round | |
| --- | --- |
| 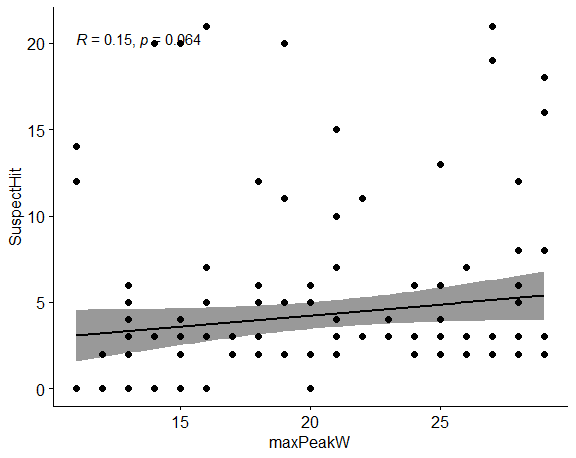 | 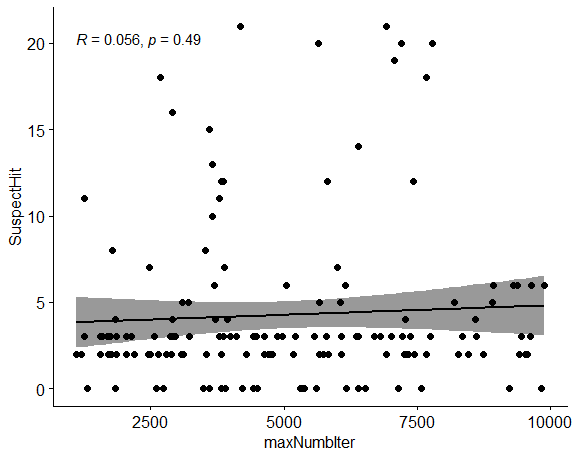 |
| 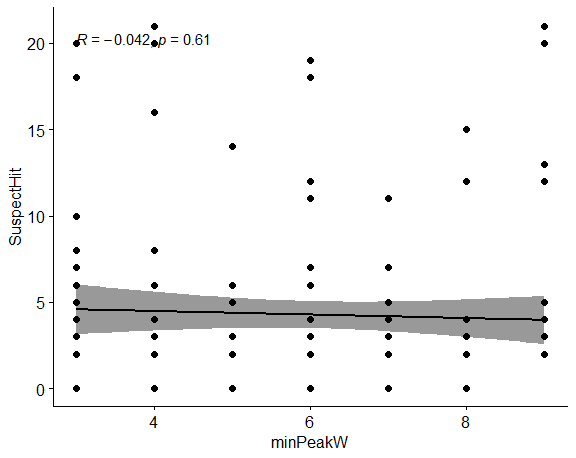 | 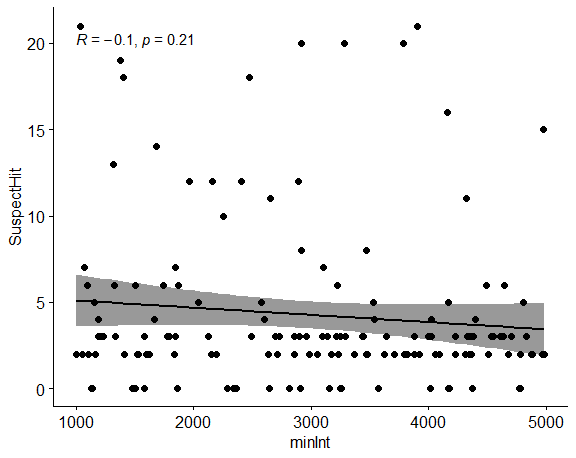 |
| 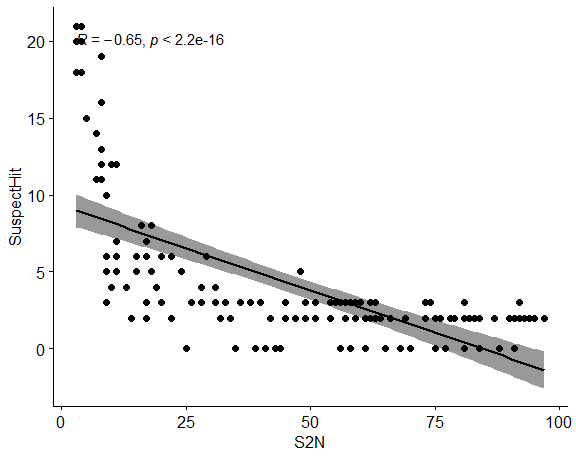 | 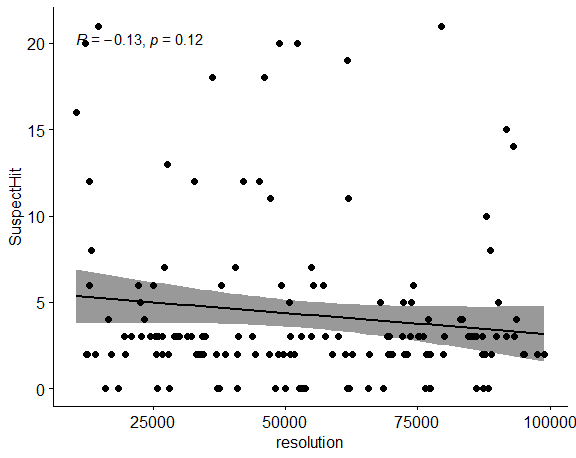 |
| SAFD - Second optimization round | |
| **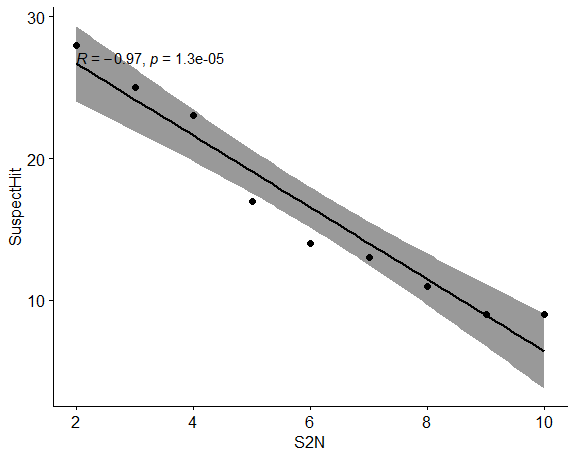** |  |

**Figure S1**. Correlation plots between the parameters of SAFD algorithms and suspect hit for first and second round.

| OpenMS- First optimization round | | |
| --- | --- | --- |
| 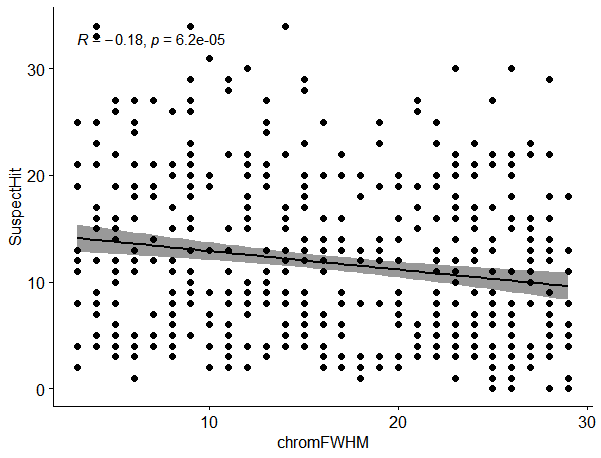 | | 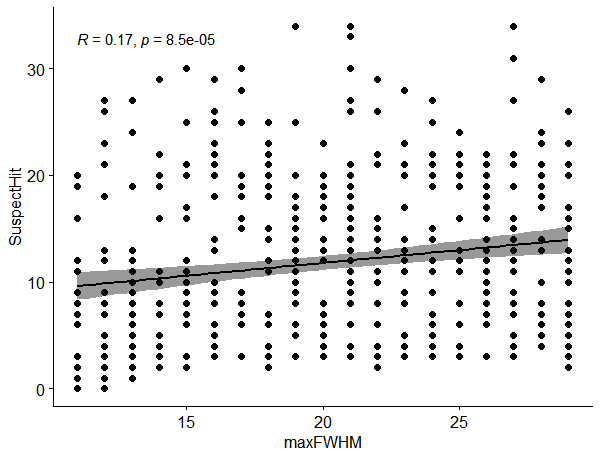 |
| 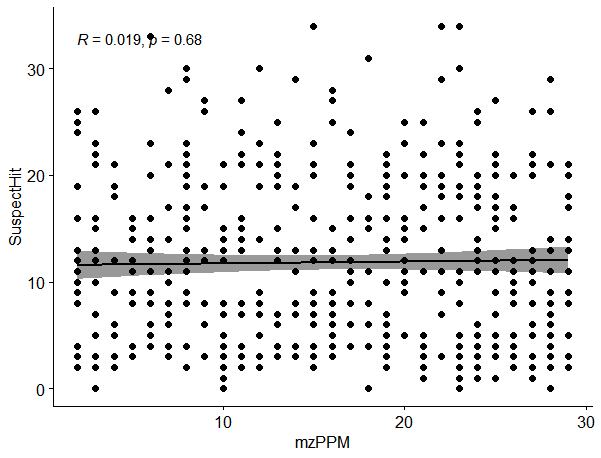 | | 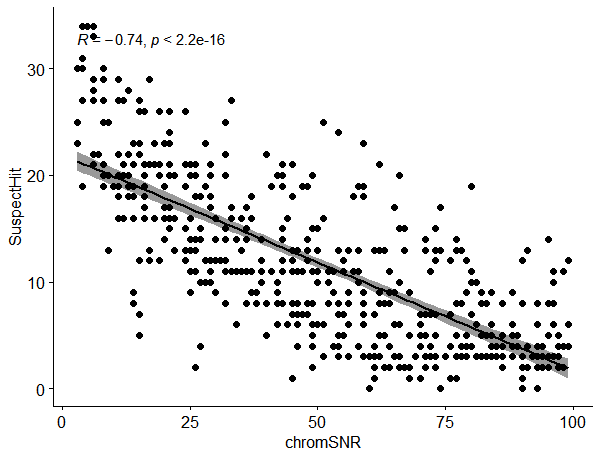 |
| 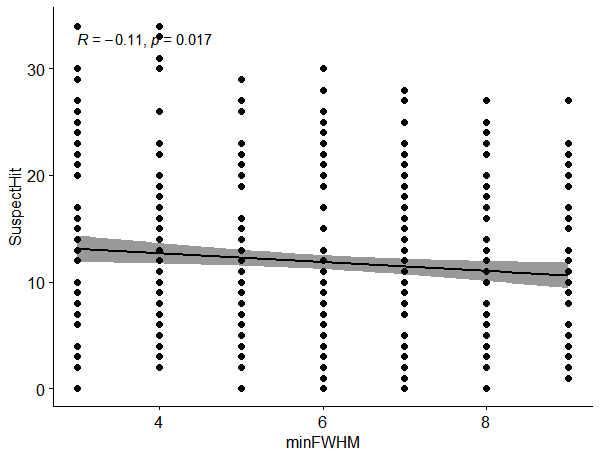 | | 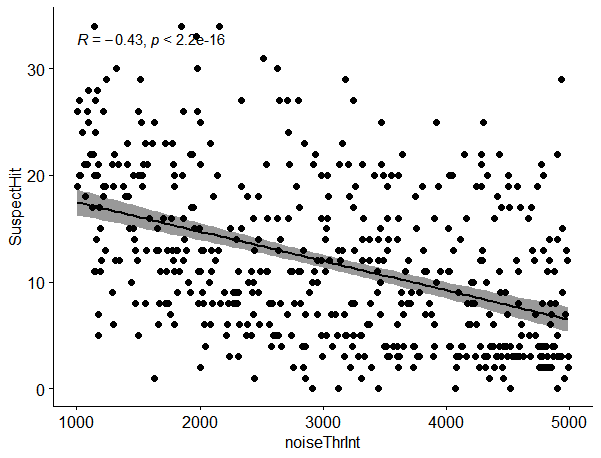 |
| OpenMS- Second optimization round | | |
| **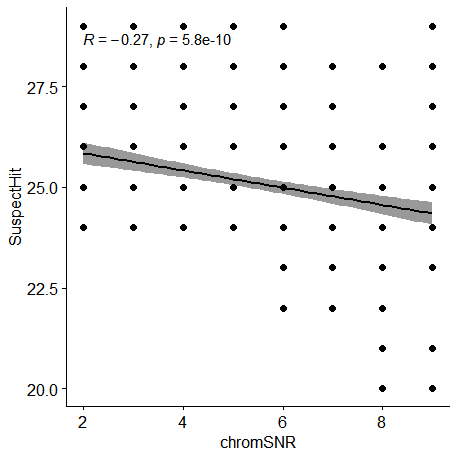** | **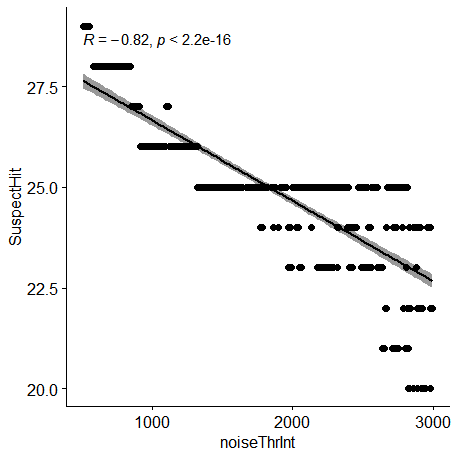** | |
| OpenMS- Third optimization round | | |
| **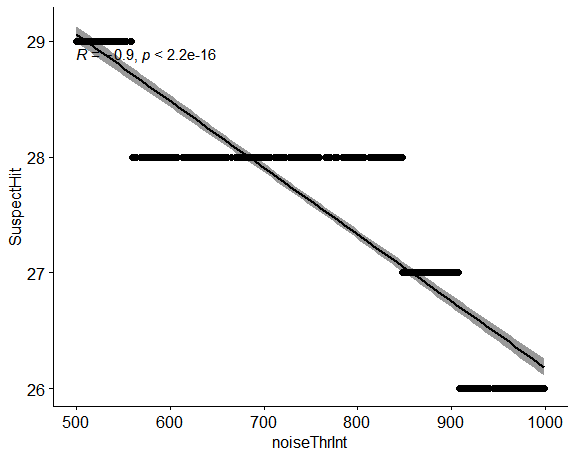** |  | |

**Figure S2**. Correlation plots between the parameters of OpenMS algorithms and suspect hit for first, second, and third round.

| XCMS - First optimization round | |
| --- | --- |
| 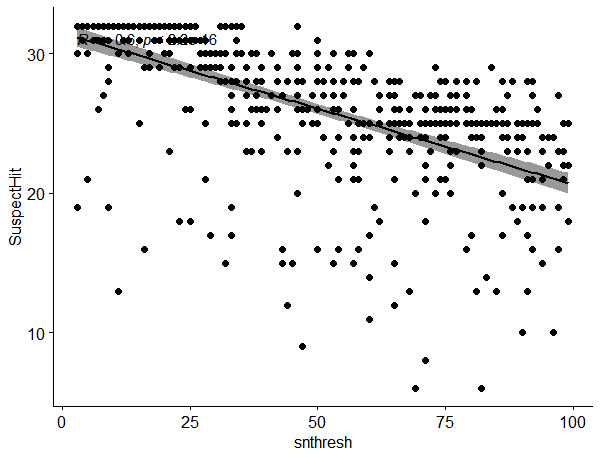 | **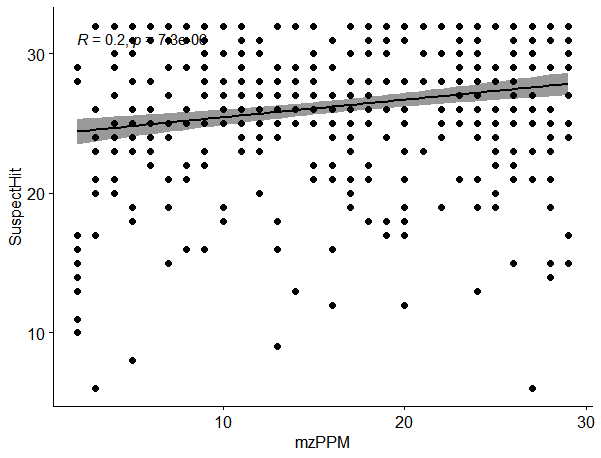** |
| 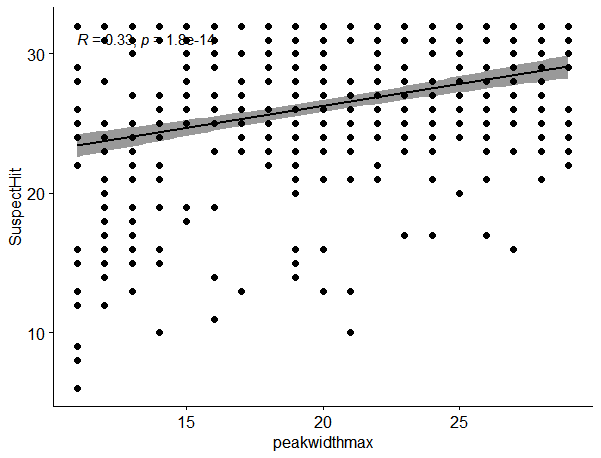 | 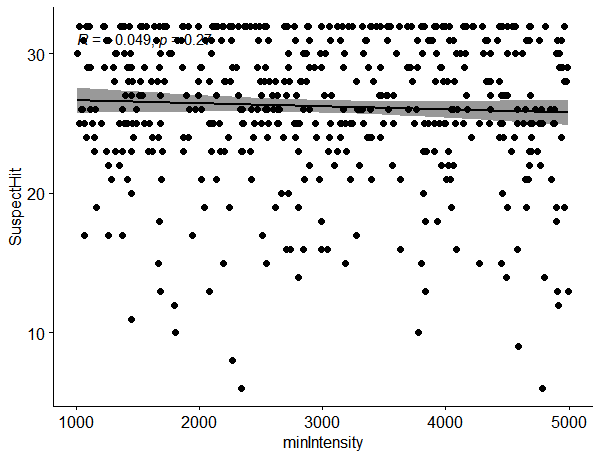 |
| **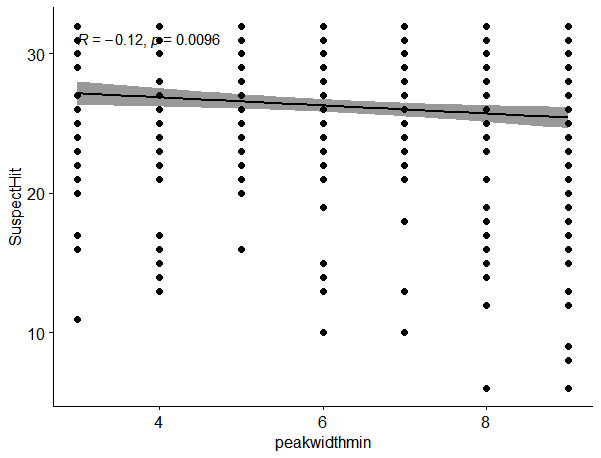** | 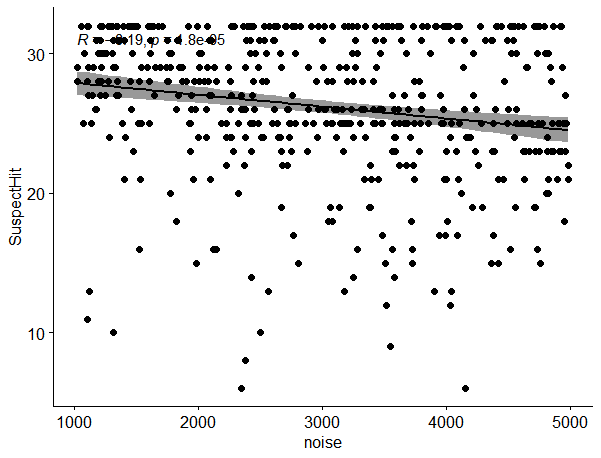 |
| XCMS - Second optimization round | |
| **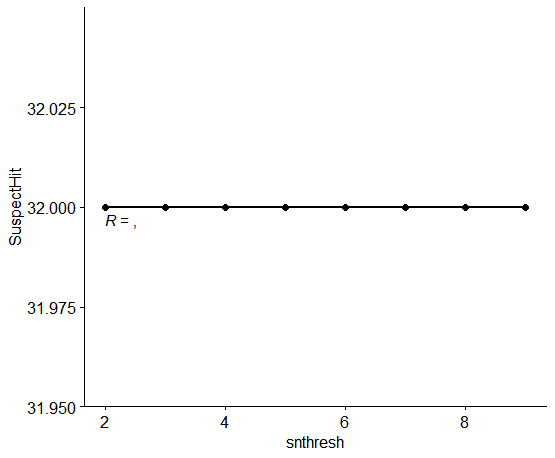** |  |

**Figure S3**. Correlation plots between the parameters of XCMS algorithms and suspect hit for first, and second round.

|  |
| --- |

| KPIC- First optimization round | |
| --- | --- |
| 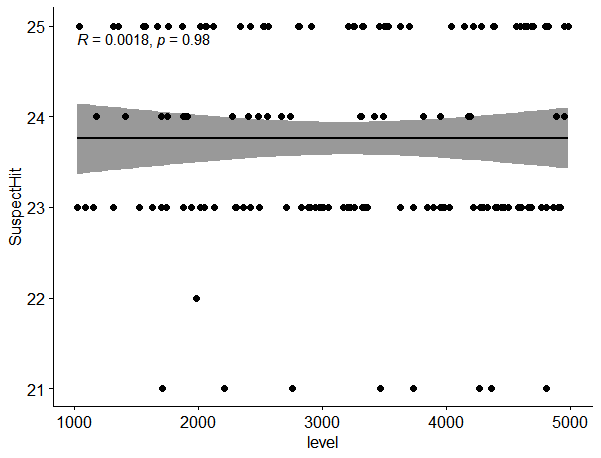 | 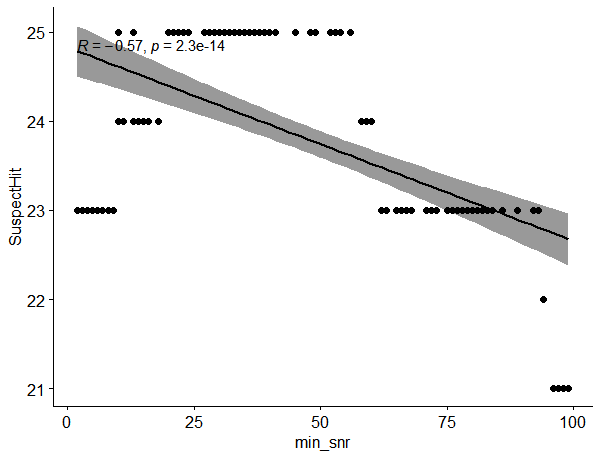 |

**Figure S4**. Correlation plots between the parameters of KPIC algorithms and suspect hit for first round.

**Correlation plots of the OpenMS feature alignment-and-grouping algorithm**

| SAFD First optimization round | |
| --- | --- |
| **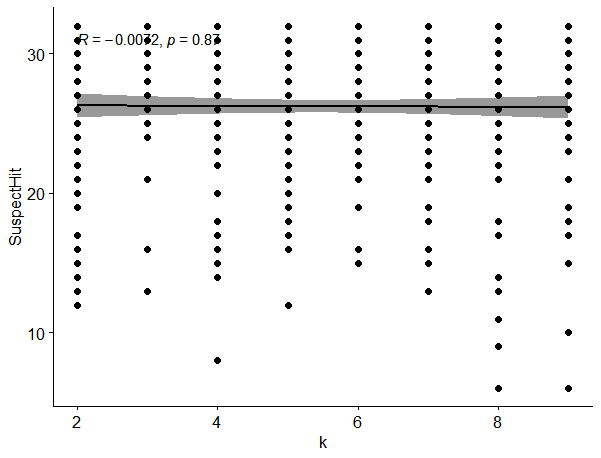** | 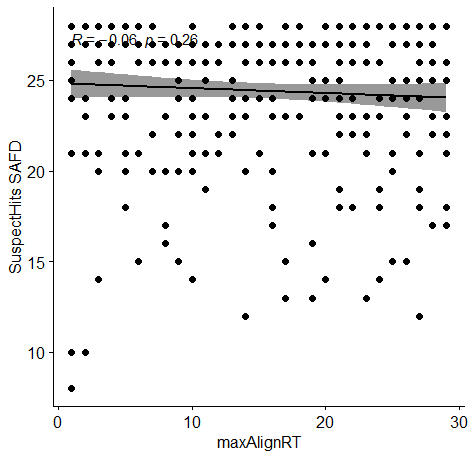 |
| 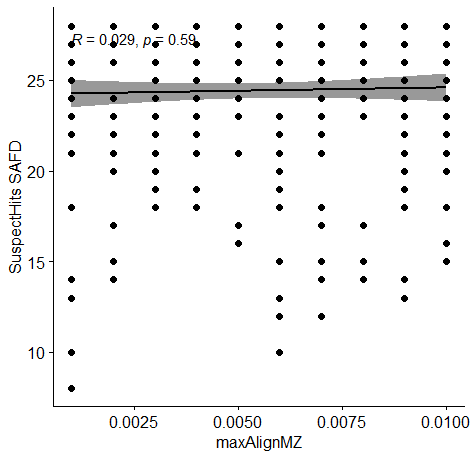 | 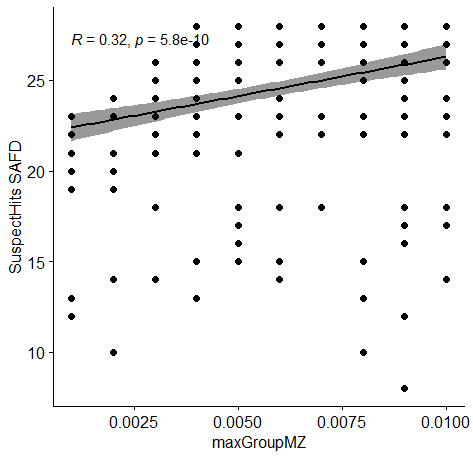 |
| 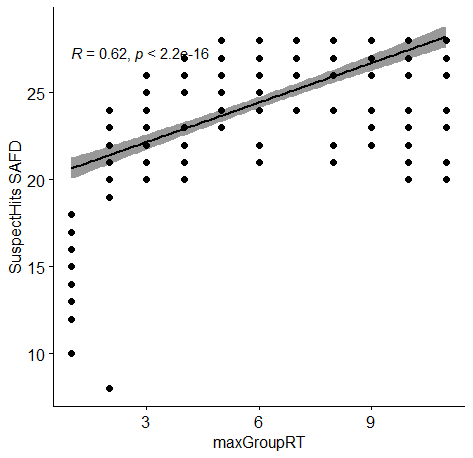 |  |
| SAFD Second optimization round | |
| 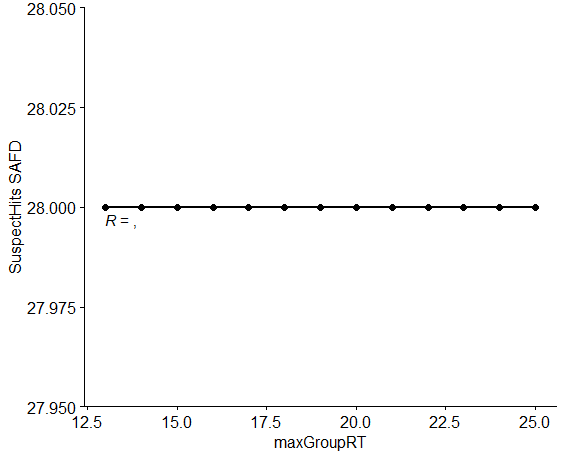 |  |

**Figure S5**. Correlation plots between the parameters of OpenMS feature alignment-and-grouping algorithm and suspect hit for first and second round in case of SAFD data.

| OpenMS First optimization round | |
| --- | --- |
| 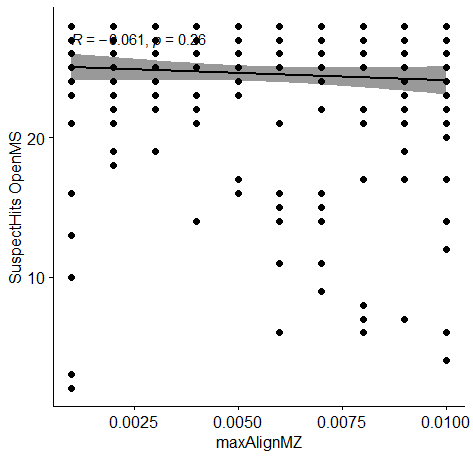 | 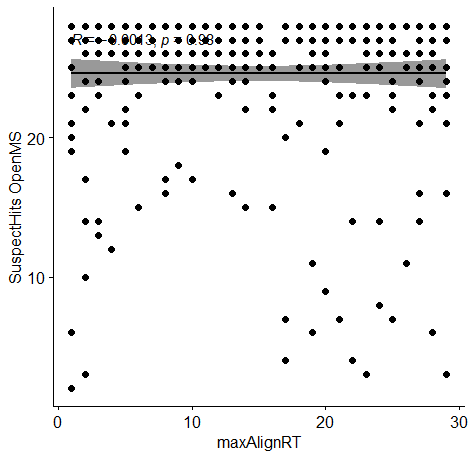 |
| 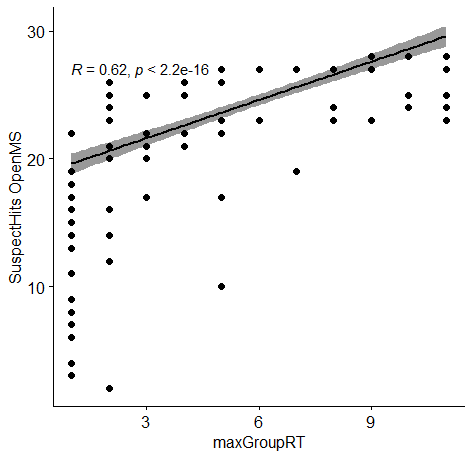 | 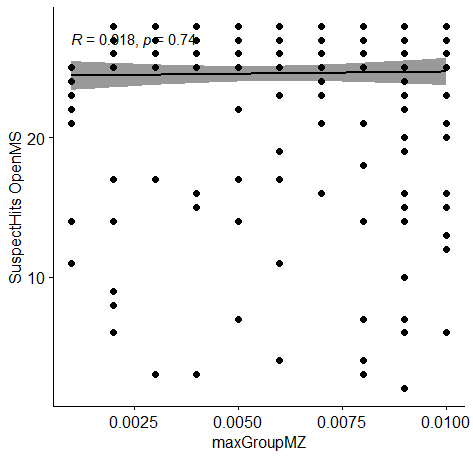 |
| OpenMS Second optimization round | |
| **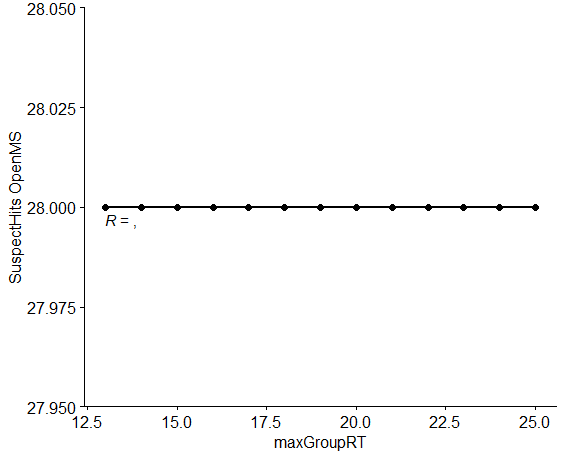** |  |

**Figure S6**. Correlation plots between the parameters of OpenMS feature alignment-and-grouping algorithm and suspect hit for first and second round in case of OpenMS data.

| XCMS First optimization round | |
| --- | --- |
| **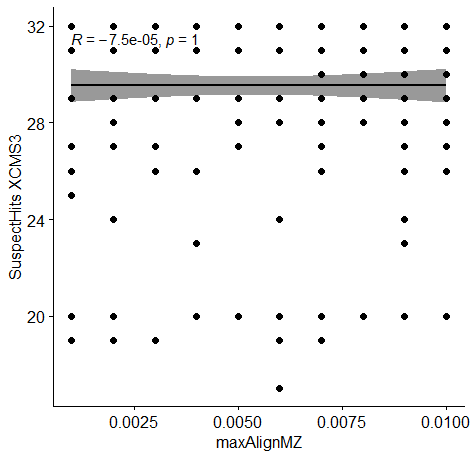** | **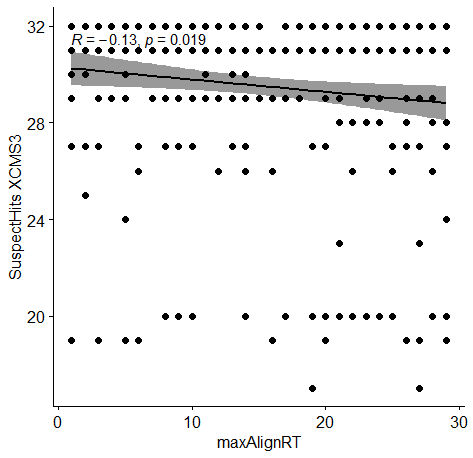** |
| **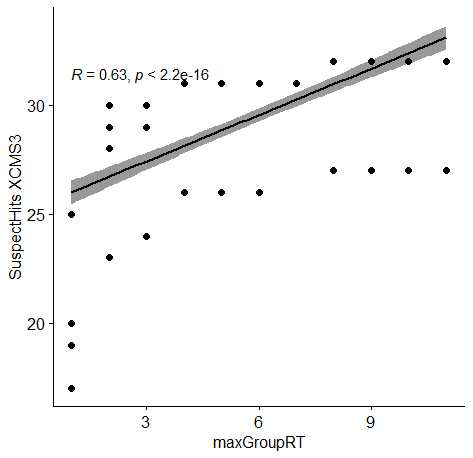** | **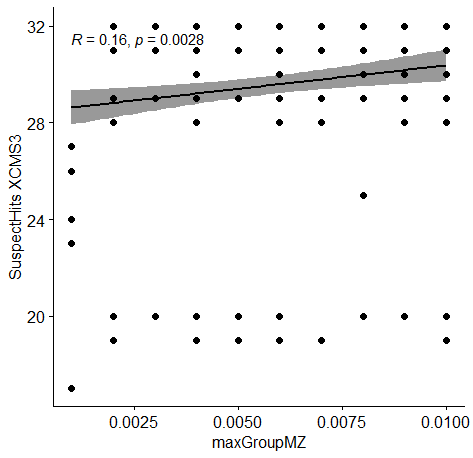** |
| XCMS Second optimization round | |
| **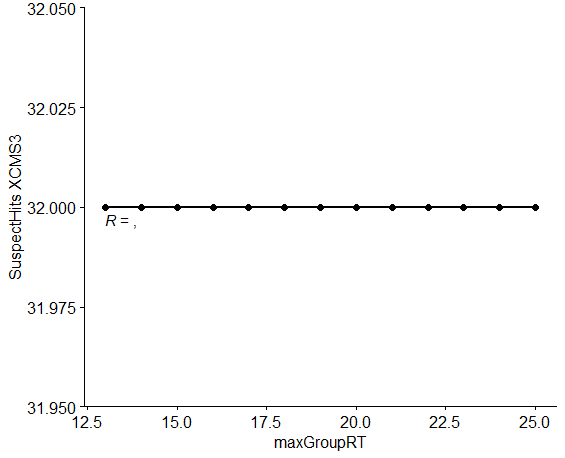** |  |

**Figure S7**. Correlation plots between the parameters of OpenMS feature alignment-and-grouping algorithm and suspect hit for first and second round in case of XCMS data.

| KPIC First optimization round | |
| --- | --- |
| **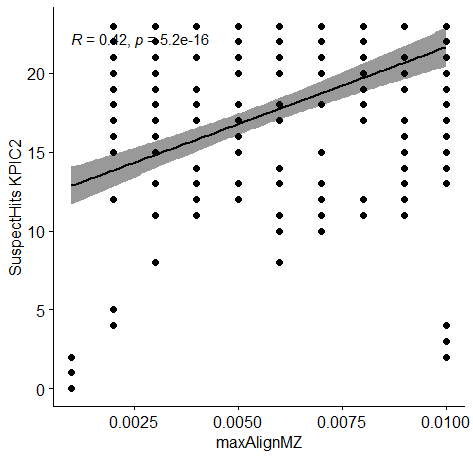** | **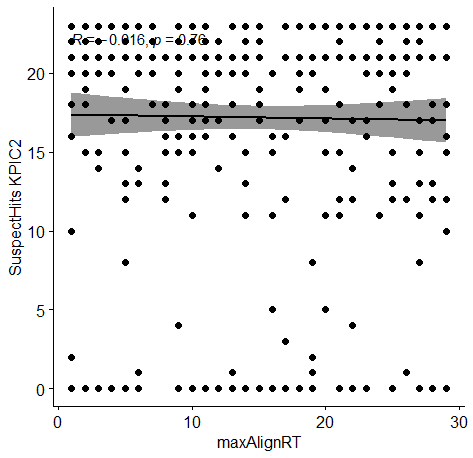** |
| **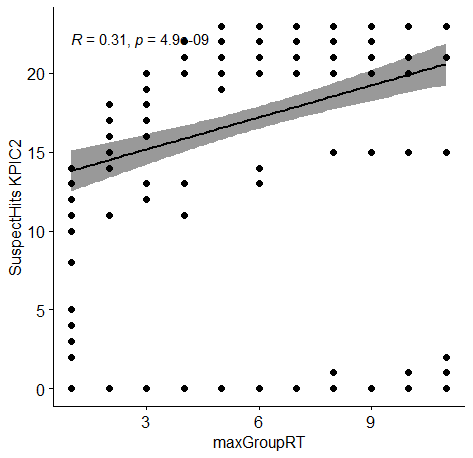** | **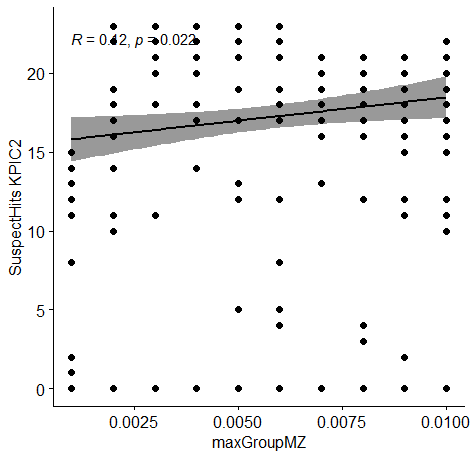** |

**Figure S8**. Correlation plots between the parameters of OpenMS feature alignment-and-grouping algorithm and suspect hit for first and second round in case of KPIC data.

# A Venn diagram for overlapping features between different algorithms using the default (DF) and optimized (OP) setting

**Using the features groups**

| **Using default parameter** | **Using optimized parameter** |
| --- | --- |
| 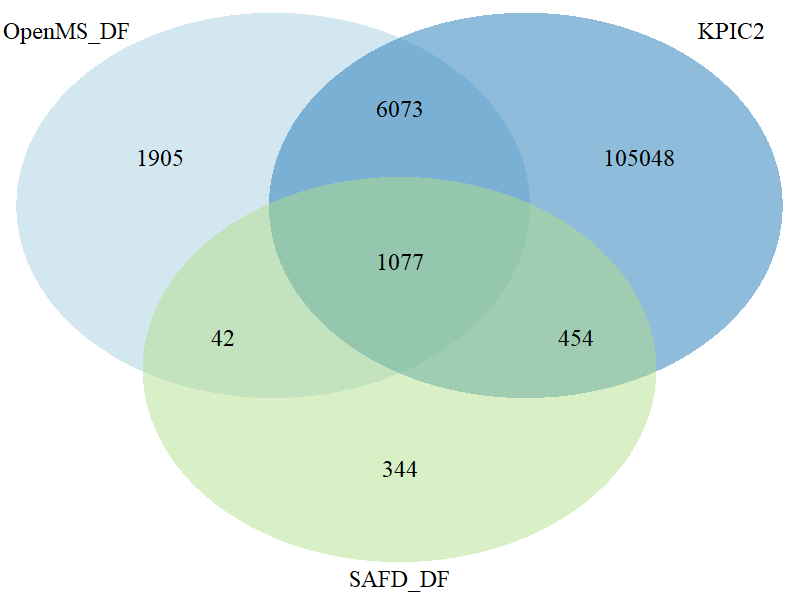 | 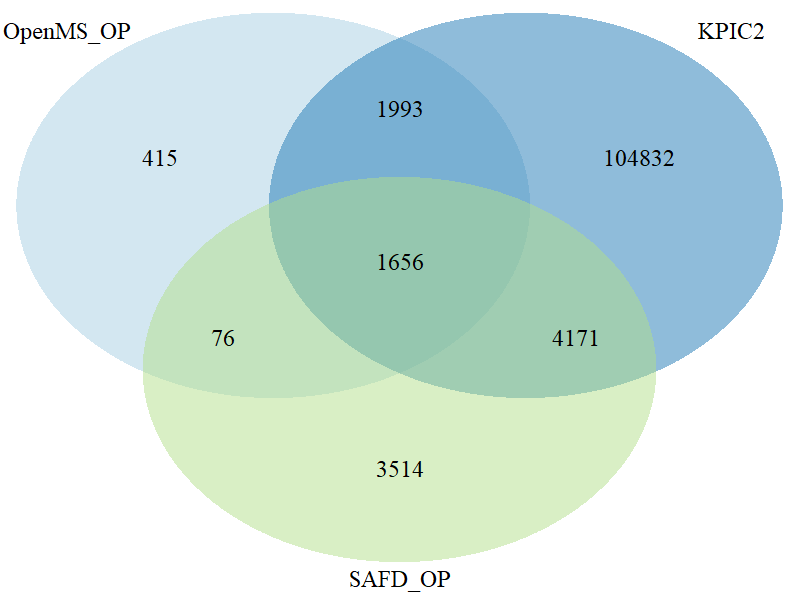 |
| 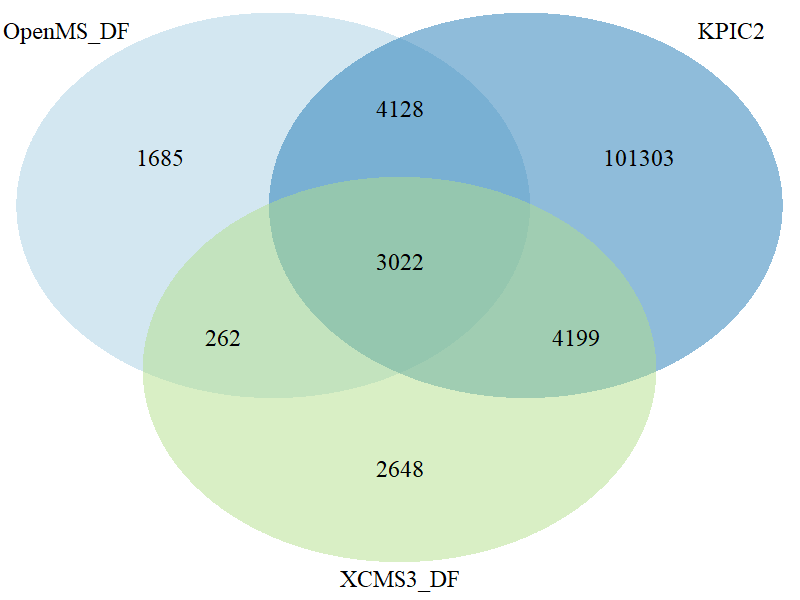 | 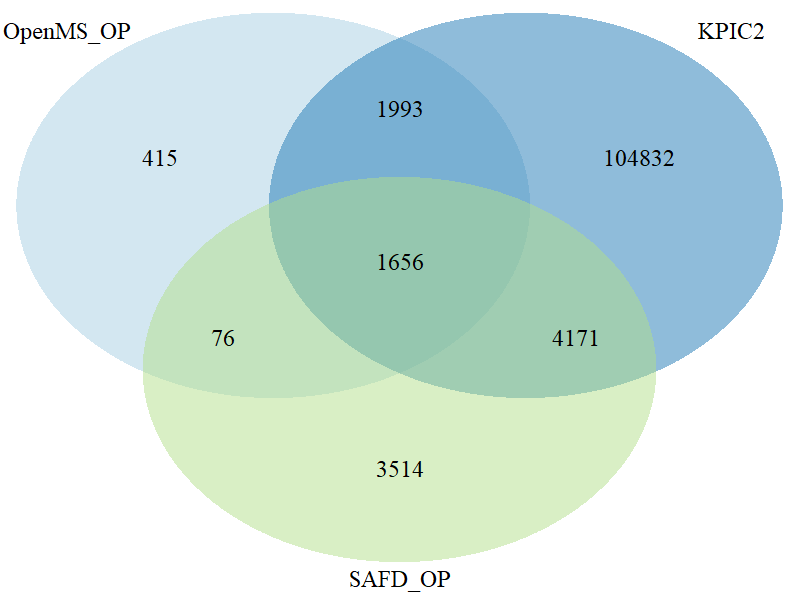 |
| 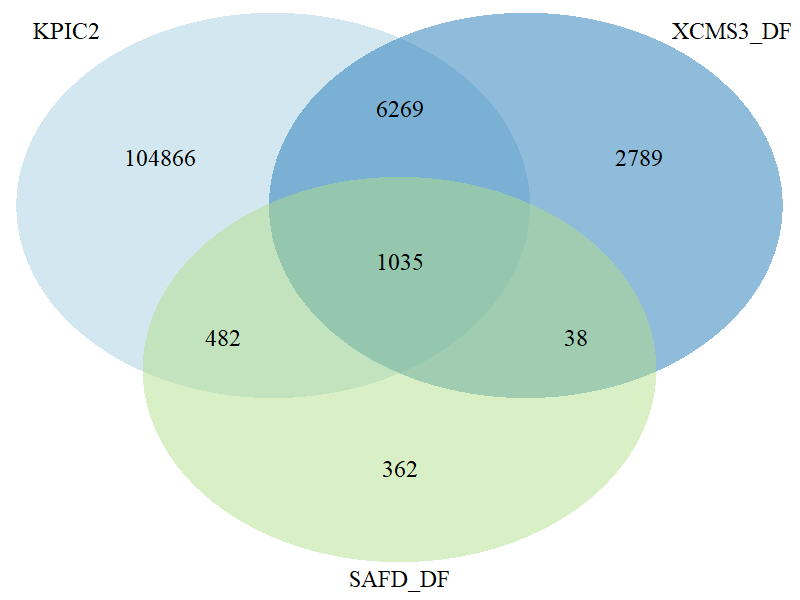 | 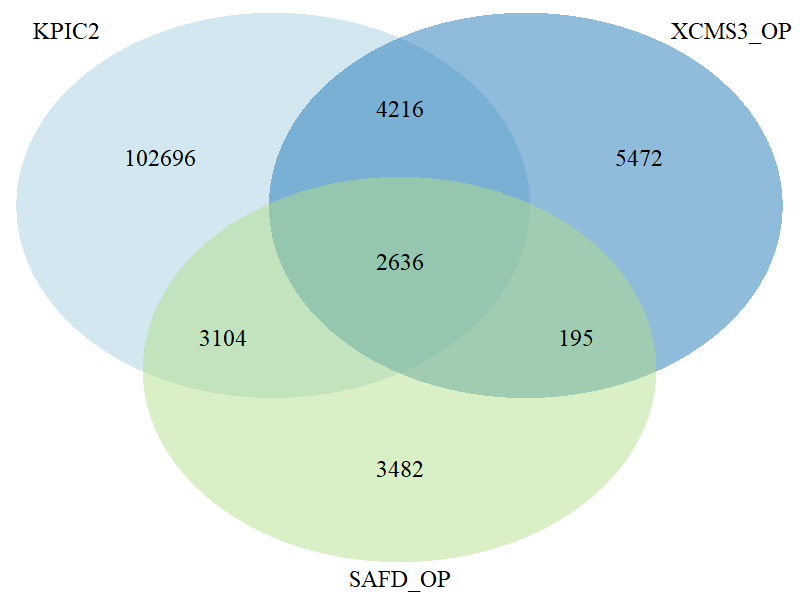 |
| 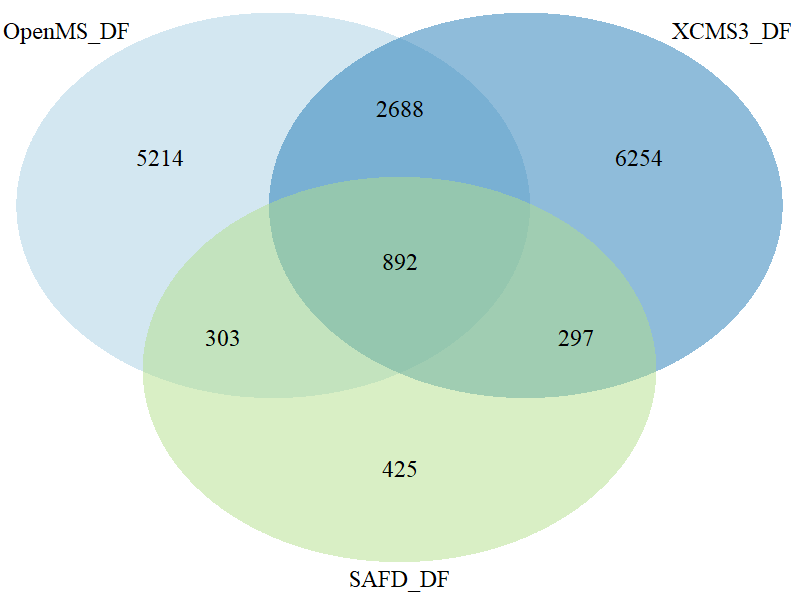 | 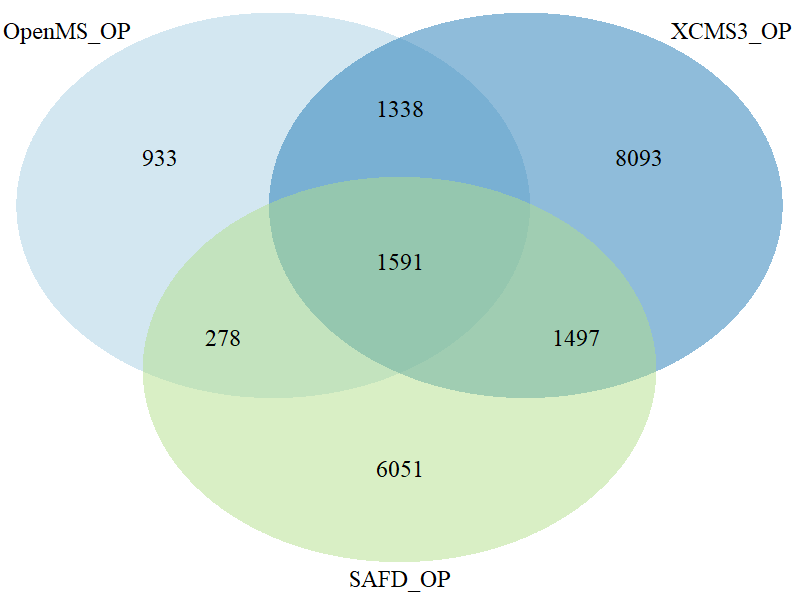 |

**Figure S9**. Venn diagram for overlapping features between three algorithms using the default (DF) and optimized (OP) setting (second scenario) using feature groups.

| 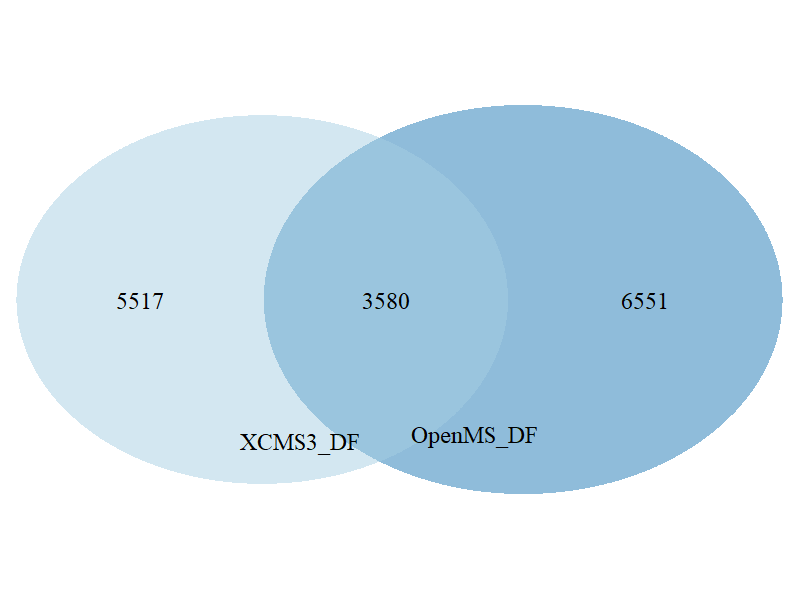 | 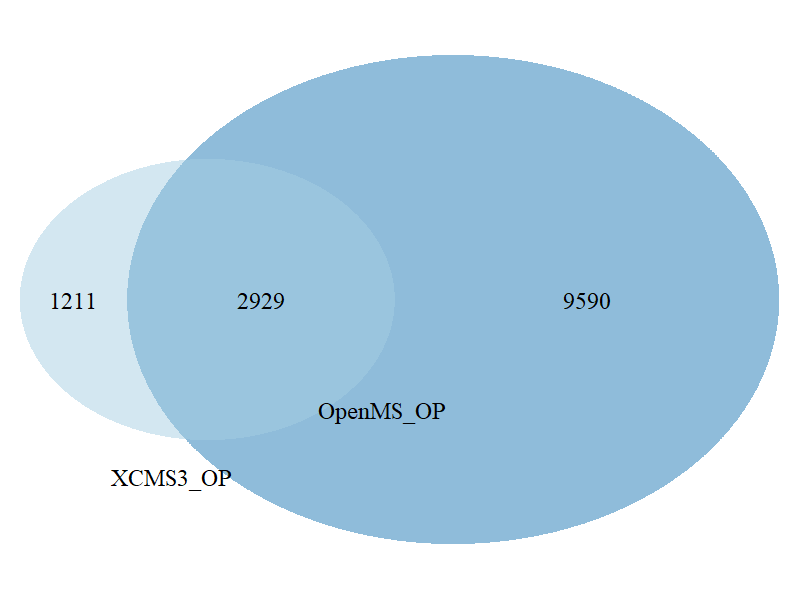 |
| --- | --- |
| 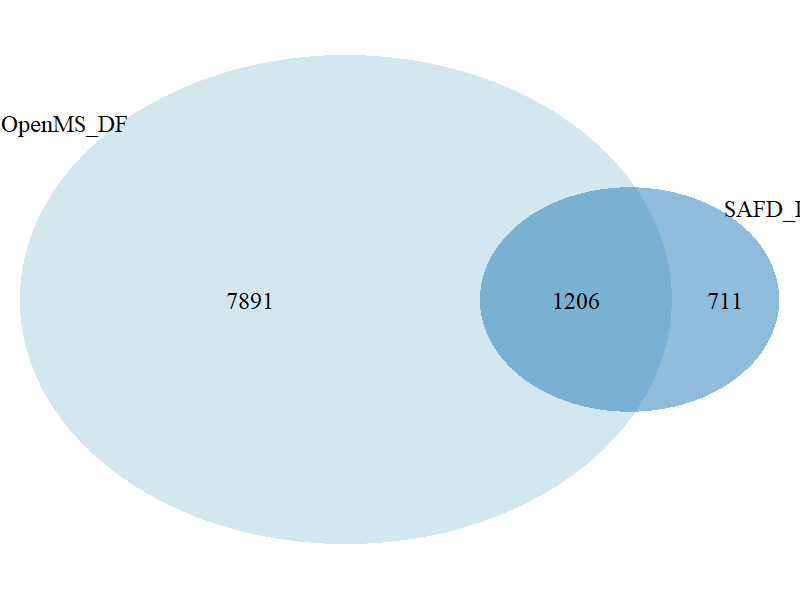 | 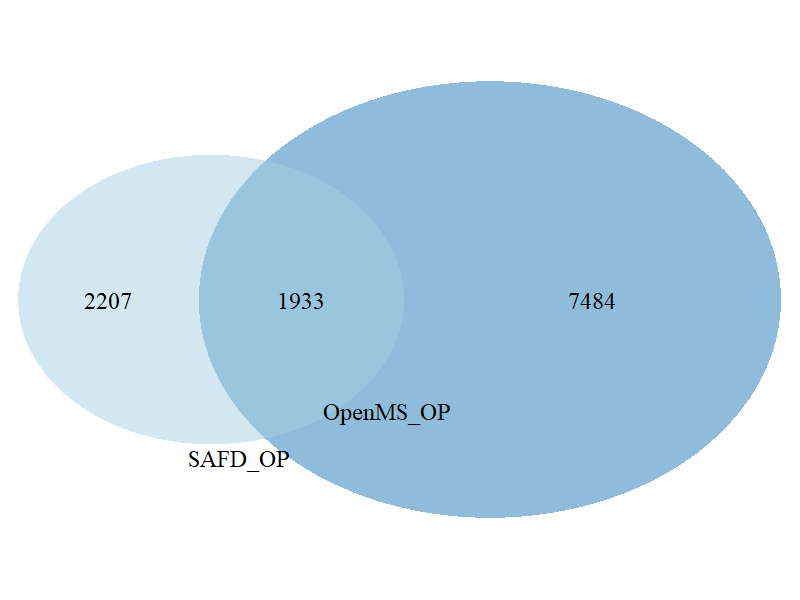 |
| 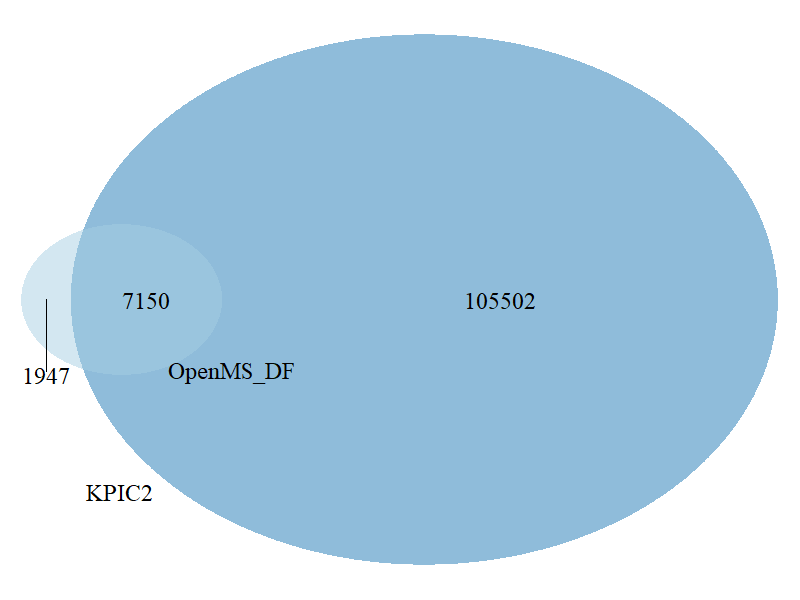 | 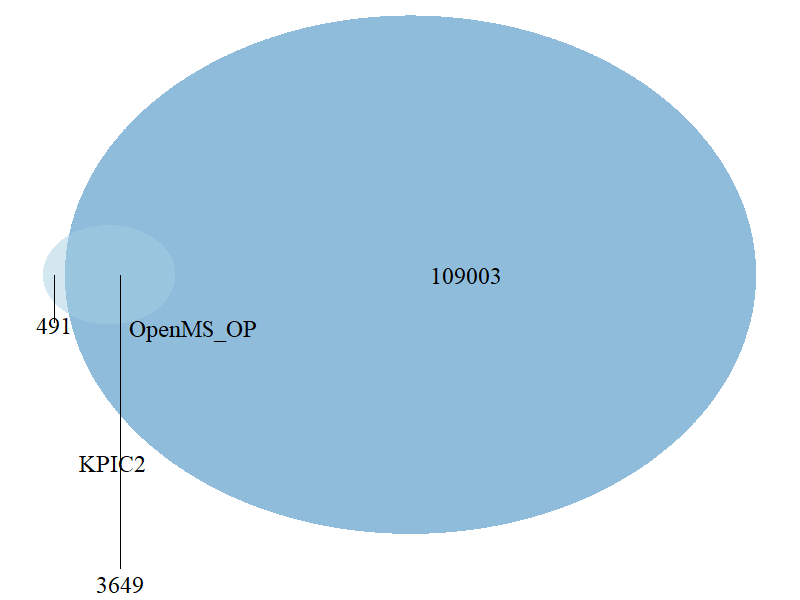 |
| 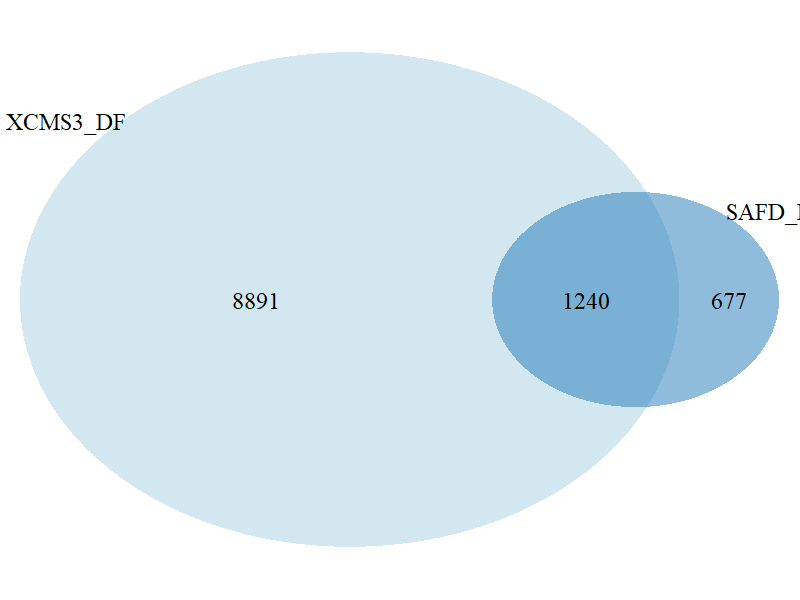 | 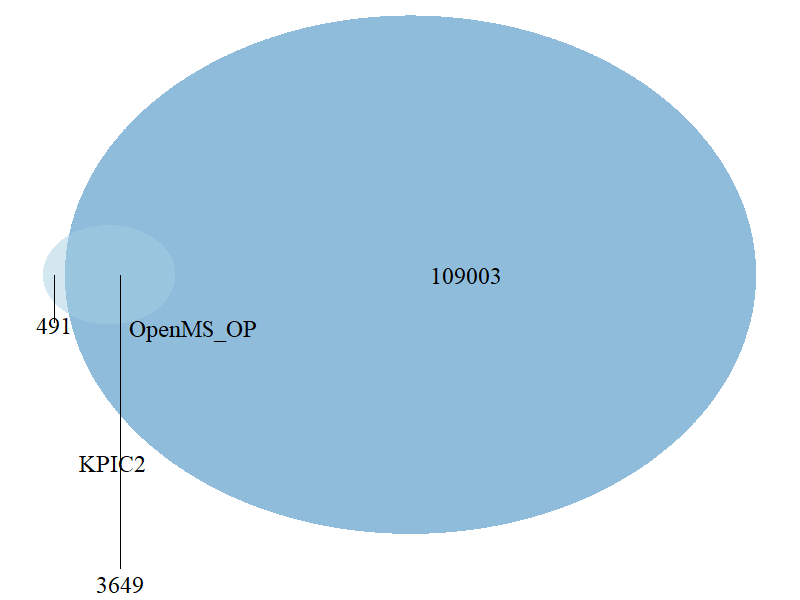 |
| 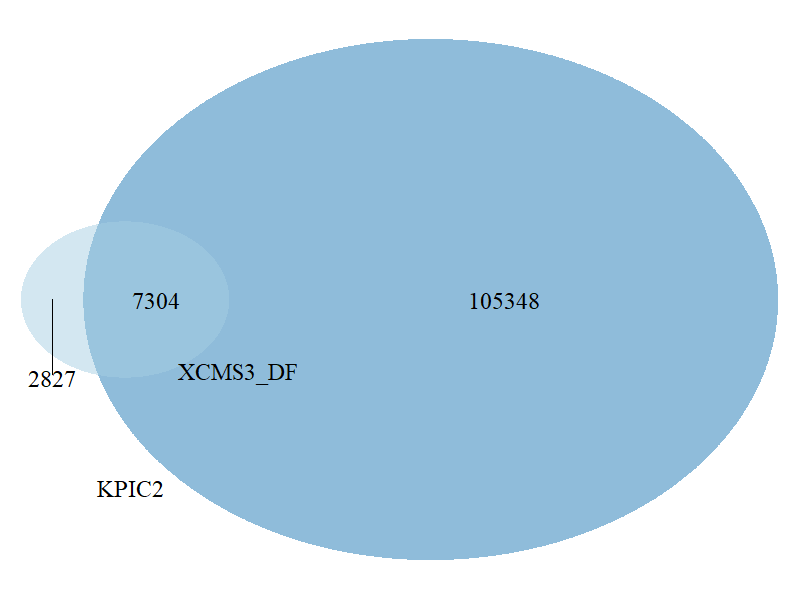 | 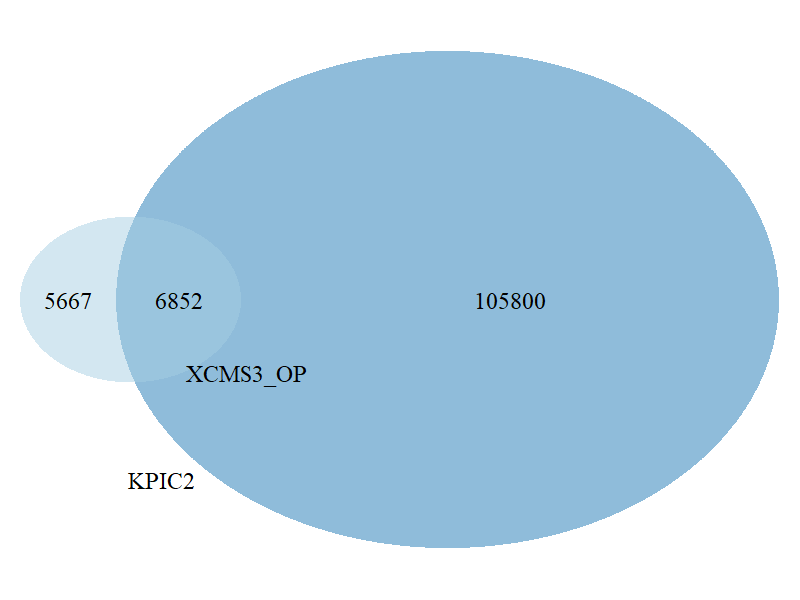 |
| 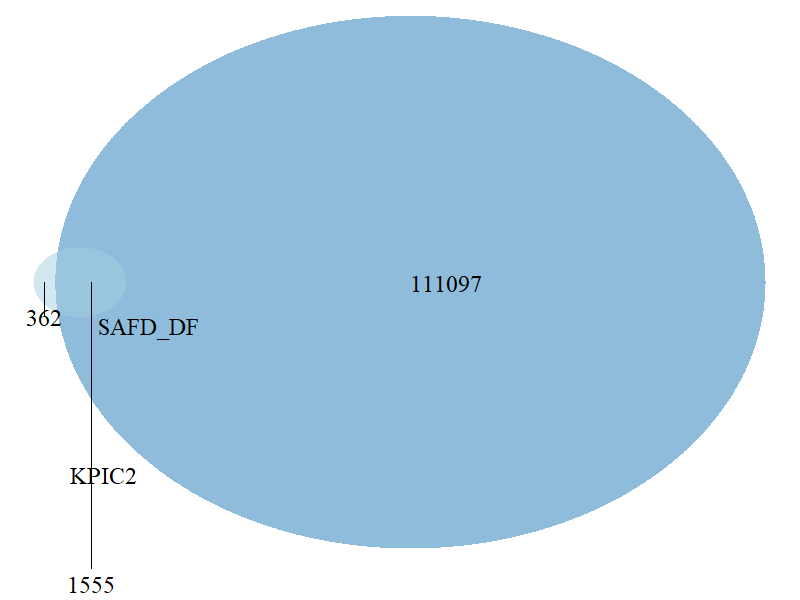 | 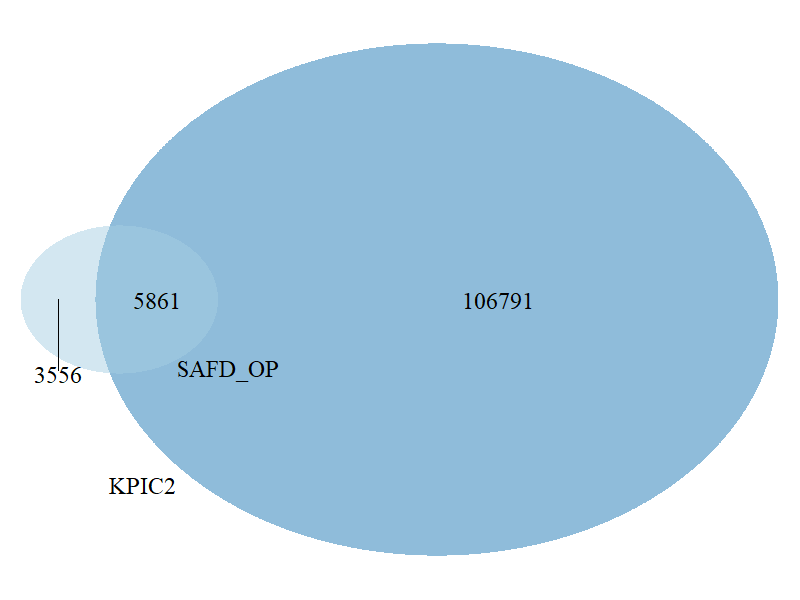 |

**Figure S10**. Venn diagram for overlapping features between two algorithms using the default (DF) and optimized (OP) setting (third scenario) using feature groups.

**Using the filtered feature groups**

| **Using default parameter** | **Using optimized parameter** |
| --- | --- |
| 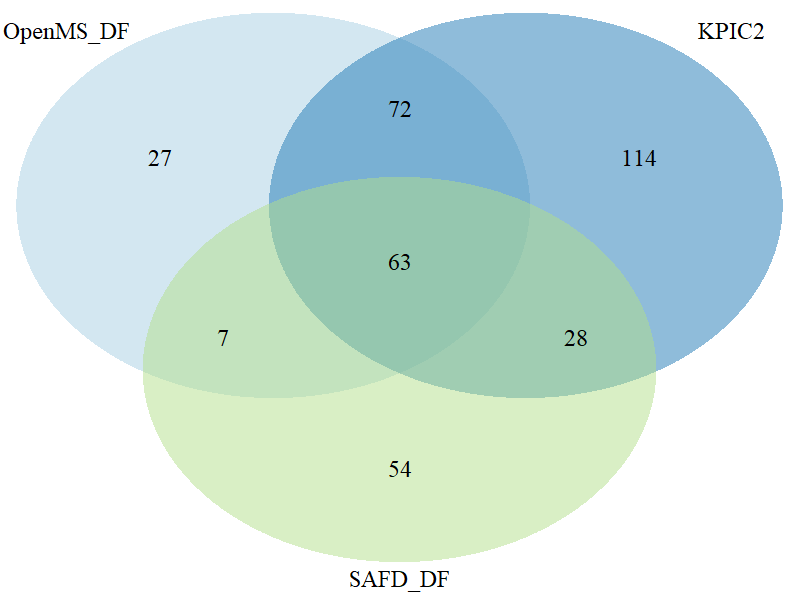 | 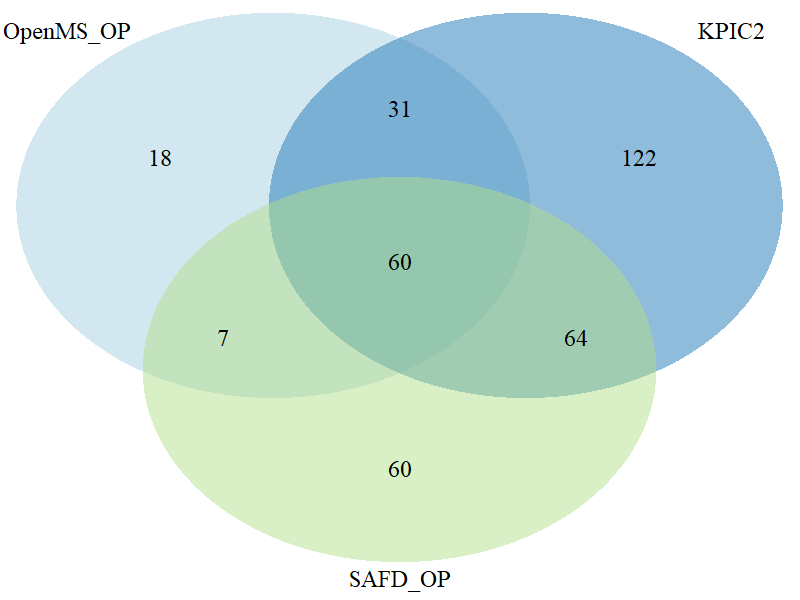 |
| 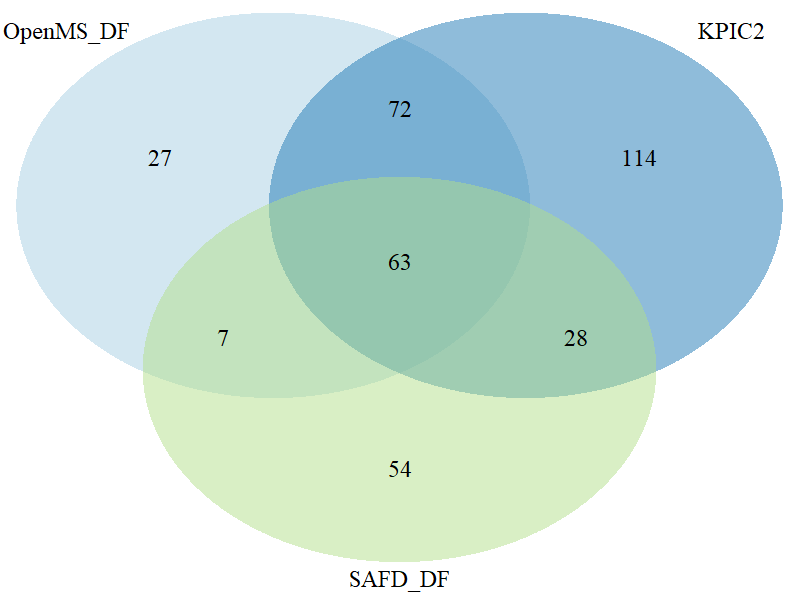 | 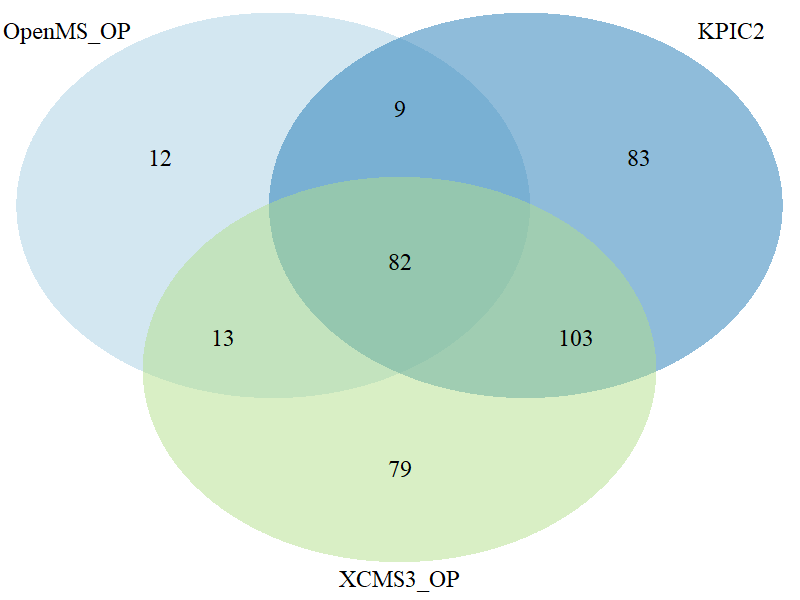 |
| 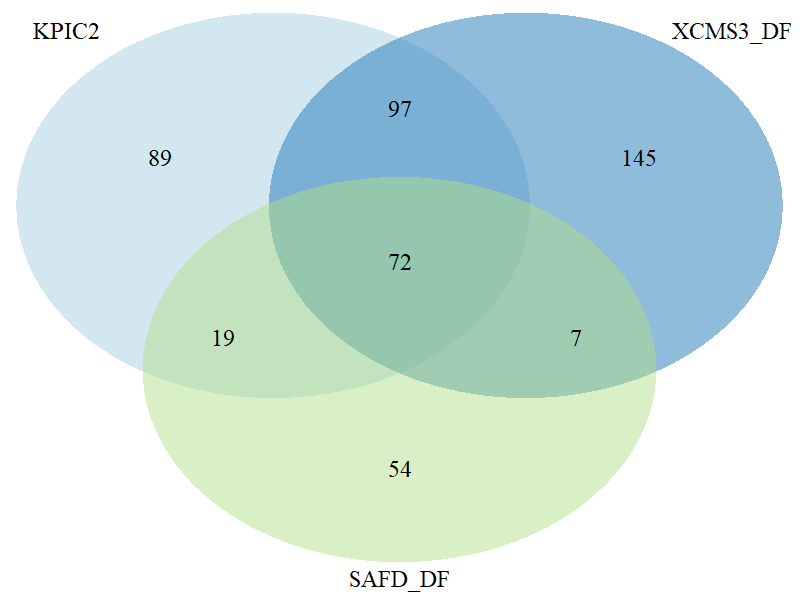 | 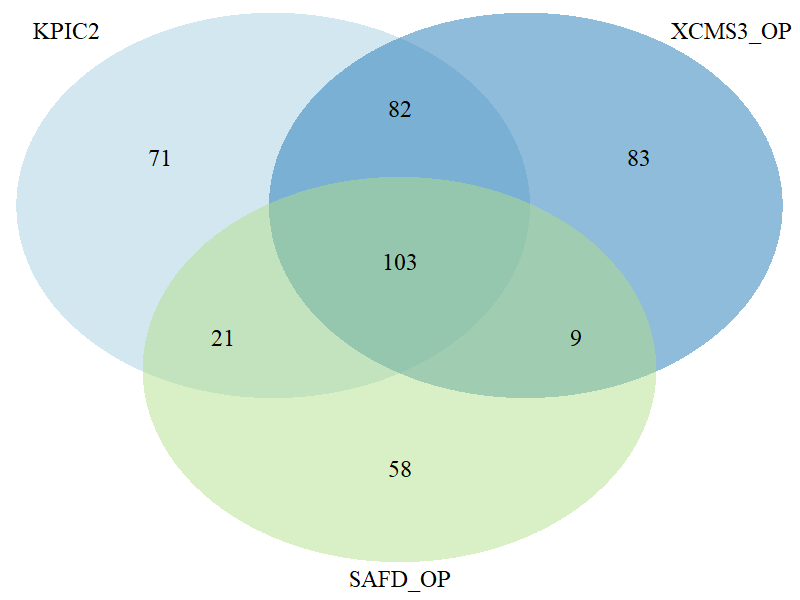 |
| 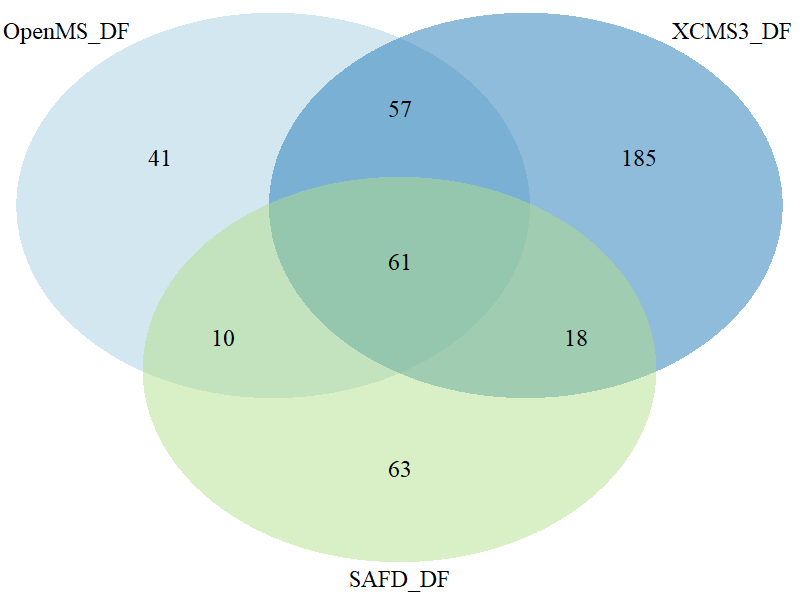 | 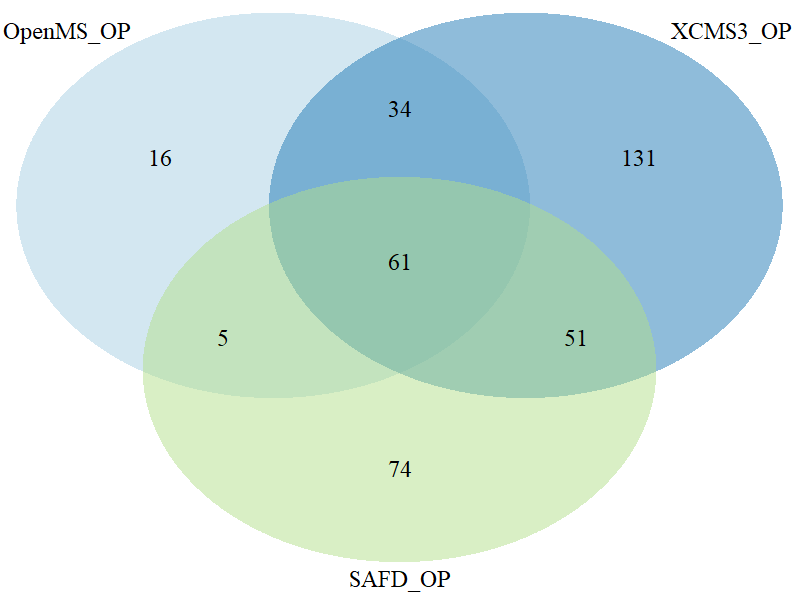 |

**Figure S11**. Venn diagram for overlapping features between three algorithms using the default (DF) and optimized (OP) setting (second scenario) using filtered feature groups.

| 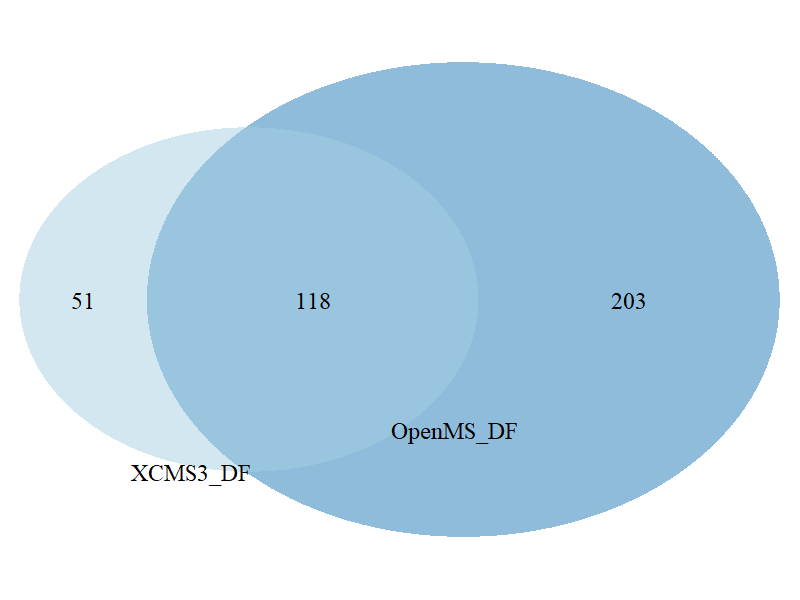 | 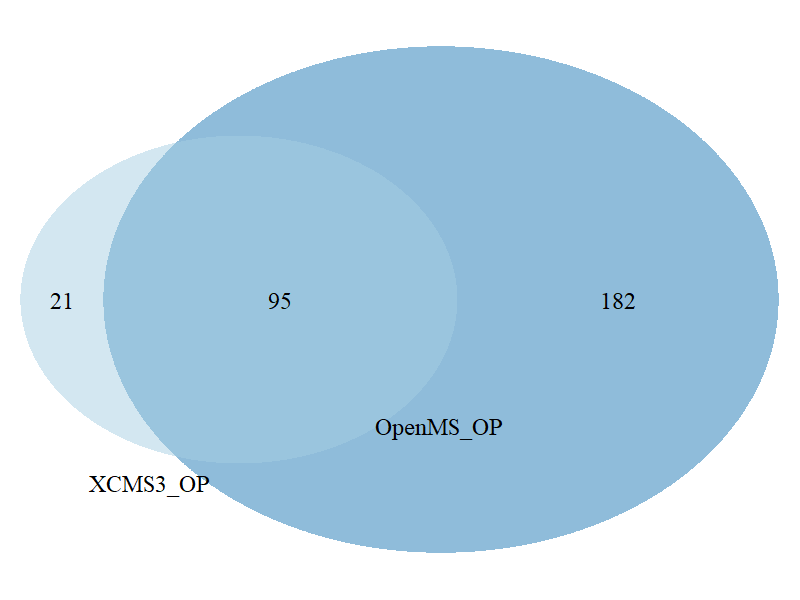 |
| --- | --- |
| 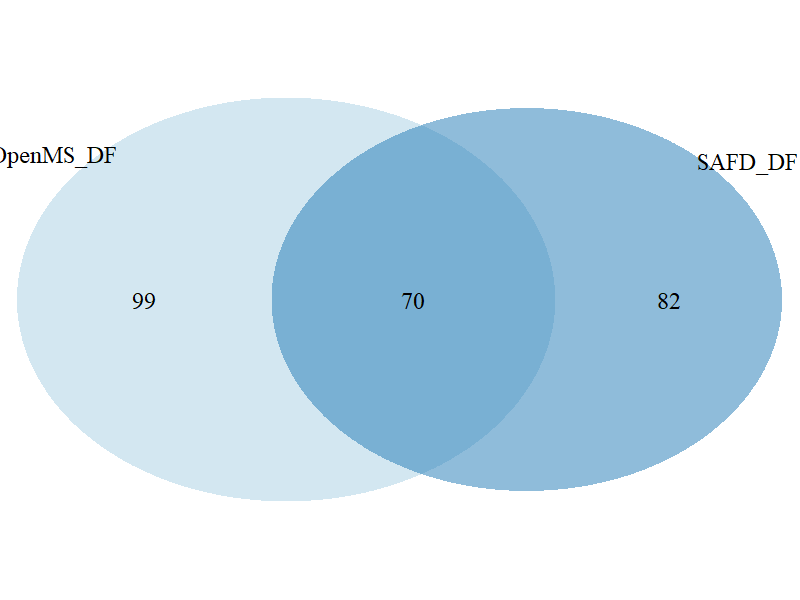 | 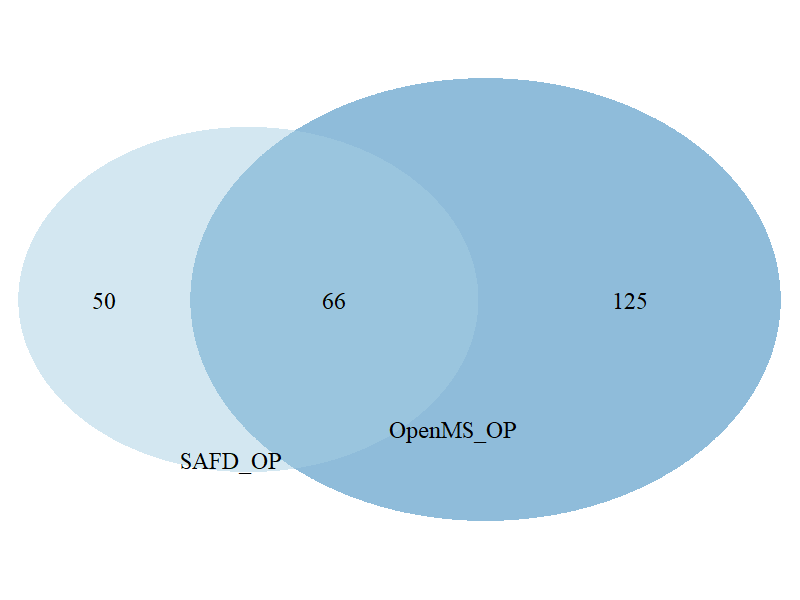 |
| 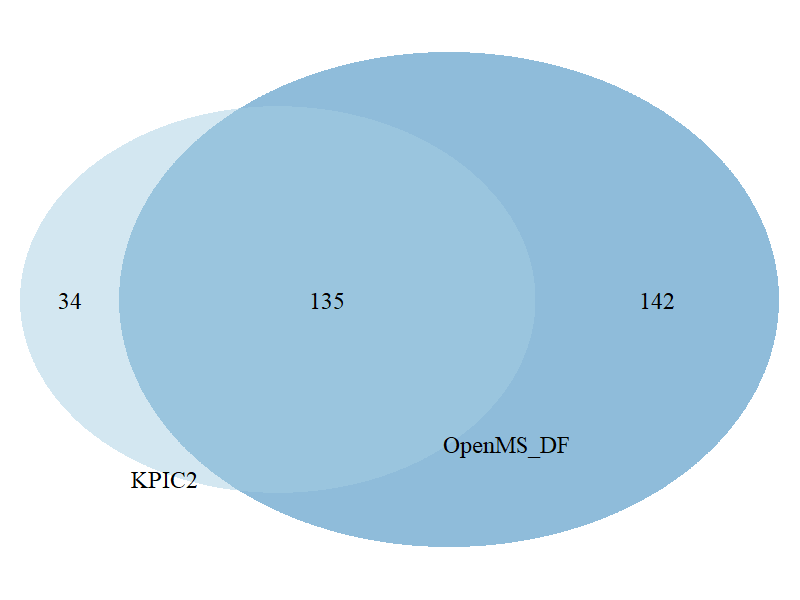 | 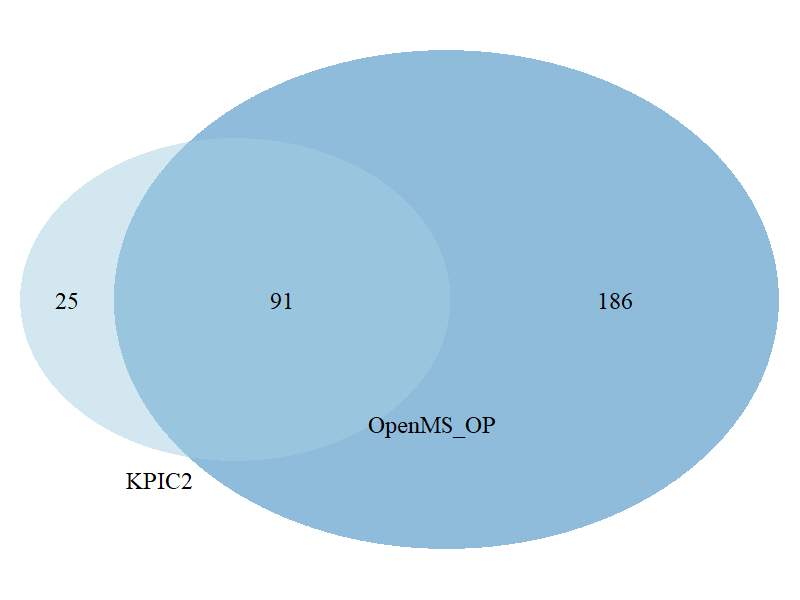 |
| 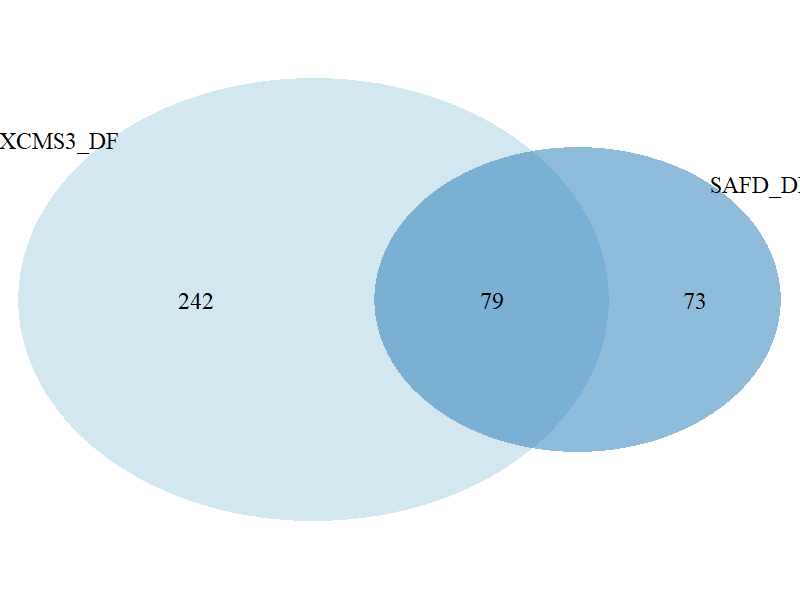 | 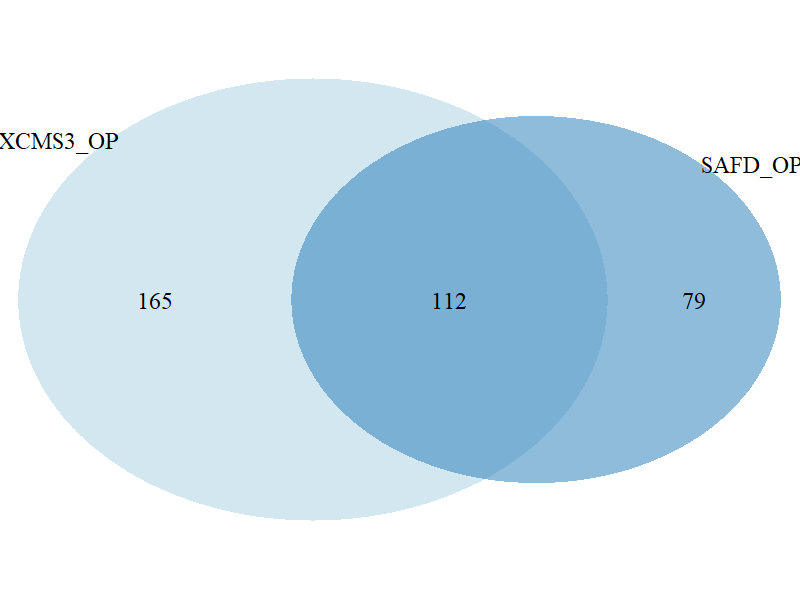 |
| 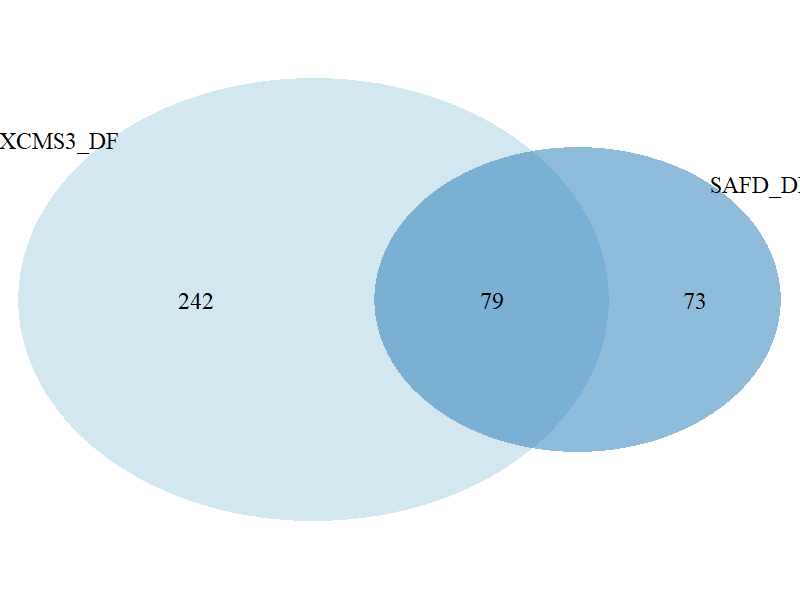 | 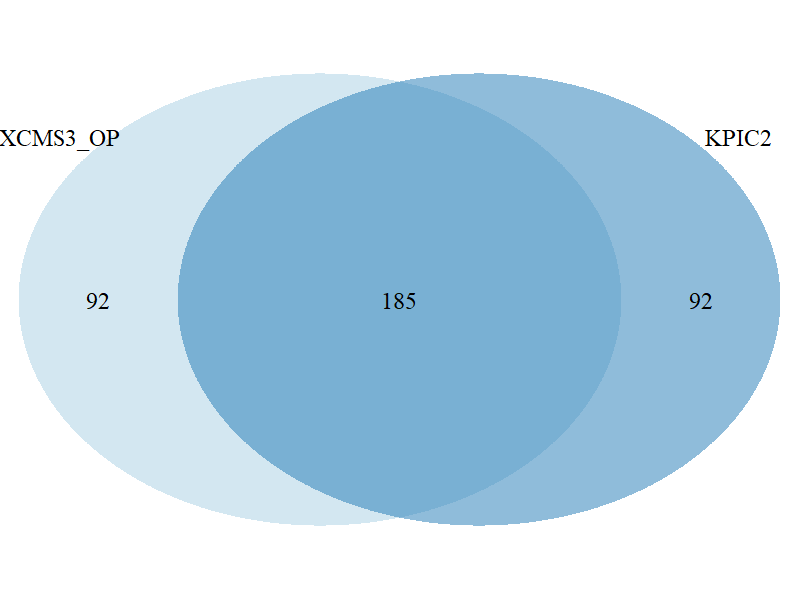 |
| 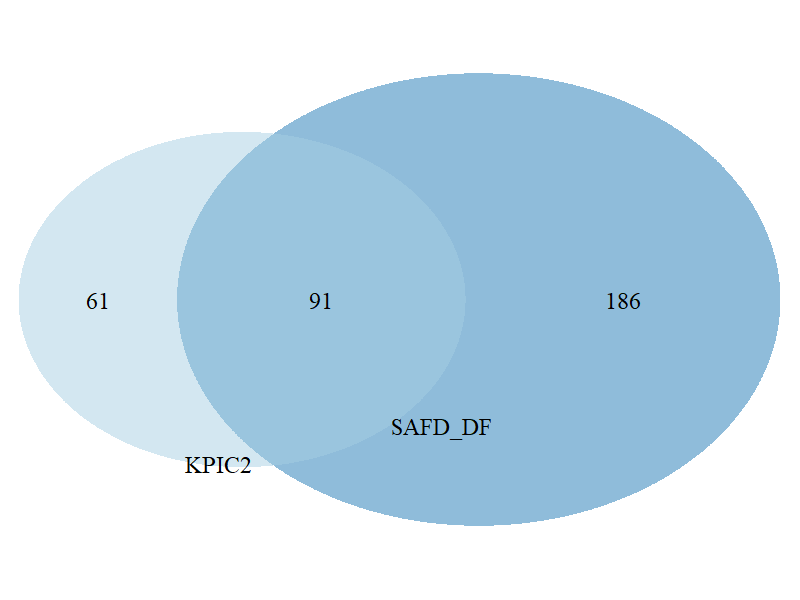 | 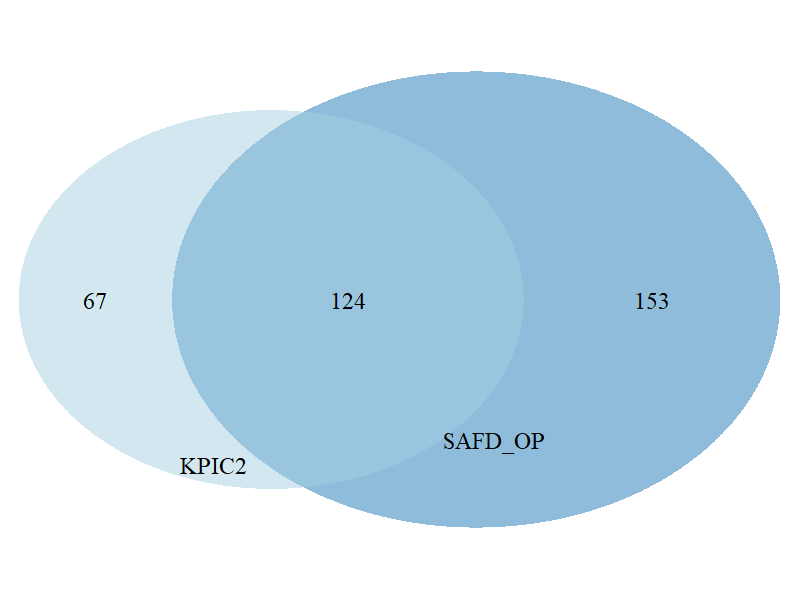 |

**Figure S12**. Venn diagram for overlapping features between two algorithms using the default (DF) and optimized (OP) setting (third scenario) using filtered feature groups.

**References**

1. Libiseller G, Dvorzak M, Kleb U, Gander E, Eisenberg T, Madeo F, Neumann S, Trausinger G, Sinner F, Pieber T, Magnes C (2015) IPO: a tool for automated optimization of XCMS parameters. BMC Bioinformatics 16:118. https://doi.org/10.1186/s12859-015-0562-8

2. Albóniga OE, González O, Alonso RM, Xu Y, Goodacre R (2020) Optimization of XCMS parameters for LC–MS metabolomics: an assessment of automated versus manual tuning and its effect on the final results. Metabolomics 16:14. https://doi.org/10.1007/s11306-020-1636-9

3. Samanipour S, O’Brien JW, Reid MJ, Thomas KV (2019) Self Adjusting Algorithm for the Nontargeted Feature Detection of High Resolution Mass Spectrometry Coupled with Liquid Chromatography Profile Data. Anal Chem 91:10800–10807. https://doi.org/10.1021/acs.analchem.9b02422

4. Röst HL, Sachsenberg T, Aiche S, Bielow C, Weisser H, Aicheler F, Andreotti S, Ehrlich H-C, Gutenbrunner P, Kenar E, Liang X, Nahnsen S, Nilse L, Pfeuffer J, Rosenberger G, Rurik M, Schmitt U, Veit J, Walzer M, Wojnar D, Wolski WE, Schilling O, Choudhary JS, Malmström L, Aebersold R, Reinert K, Kohlbacher O (2016) OpenMS: a flexible open-source software platform for mass spectrometry data analysis. Nature Methods 13:741–748. https://doi.org/10.1038/nmeth.3959

5. Benton HP, Wong DM, Trauger SA, Siuzdak G (2008) XCMS2: Processing Tandem Mass Spectrometry Data for Metabolite Identification and Structural Characterization. Anal Chem 80:6382–6389. https://doi.org/10.1021/ac800795f

6. Ji H, Zeng F, Xu Y, Lu H, Zhang Z (2017) KPIC2: An Effective Framework for Mass Spectrometry-Based Metabolomics Using Pure Ion Chromatograms. Anal Chem 89:7631–7640. https://doi.org/10.1021/acs.analchem.7b01547
